# Weak Anion Hydration Drives LCST Behavior in Ionic Liquid–Water Mixtures: Insights from Calorimetry and Neutron Diffraction

**DOI:** 10.1021/acs.jpcb.6c04727

**Published:** 2026-10-11

**Authors:** Sanskrita Madhukailya, Guillaume Depraetère, Nithavong Cam, Tristan G. A. Youngs, James P. Tellam, Margarida Costa Gomes, Leila Moura, John D. Holbrey

**Affiliations:** † The QUILL Research Centre, School of Chemistry and Chemical Engineering, 1596 Queen’s University Belfast, David Keir Building, Stranmillis Road, Belfast BT9 5AG, U.K.; ‡ 26911 Laboratoire de Chimie, ENS Lyon, CNRS and Université de Lyon, 46 allée d’Italie, 69364 Lyon, France; § ISIS Neutron and Muon Source, Rutherford Appleton Laboratory, 55135 Harwell Science & Innovation Campus, Didcot, Oxfordshire OX11 0QX, U.K.

## Abstract

Isothermal titration calorimetry (ITC) combined with neutron diffraction and isotopic substitution (NDIS) has been used to elucidate the molecular origins of lower critical solution temperature (LCST) behavior in the tetrabutylphosphonium 5-phenyltetrazolate/water ([P_4444_]­[Ph–tet]/H_2_O) system. Comparison with the structurally similar but fully water-miscible analogue, tetrabutylphosphonium benzoate ([P_4444_]­[Bz]) reveals a much smaller negative enthalpy of mixing with water. This is indicative that weaker solvation of the [Ph–tet]^−^ anion drives an entropy-dominated desolvation process upon heating, leading to phase separation into IL-rich and water-rich domains and is supported by observation from NDIS that the [Ph–tet]^−^ anion is only weakly hydrated by water.

## Graphical abstract

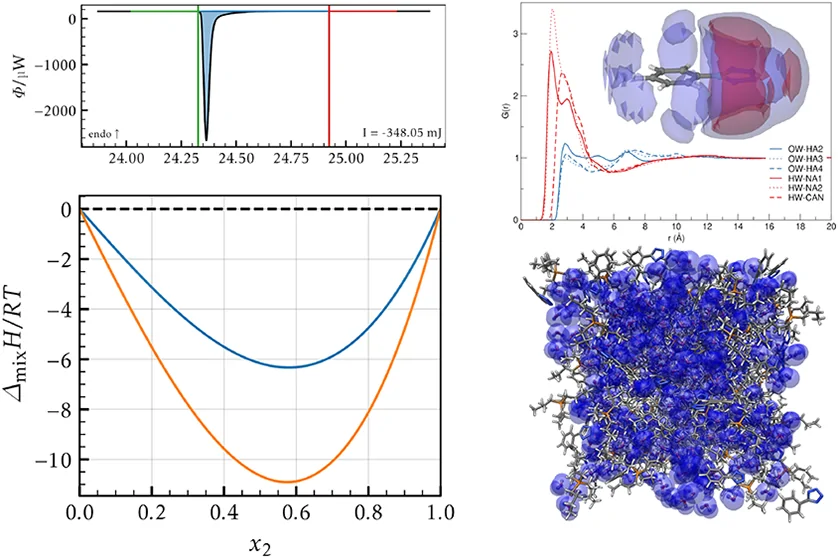

## Introduction

Hydrophobic ionic liquids (ILs)—those that form biphasic mixtures with water—have been of interest as replacements for volatile organic solvents in aqueous/organic liquid–liquid separations for a considerable time.^1^ Beyond intrinsically hydrophobic ILs that form permanent biphases with water across its normal liquid range, many ILs exhibit temperature- or composition-dependent miscibility, giving rise to a rich variety of phase behaviors. These include (i) biphase formation driven by the inherent hydrophobicity of the cation–anion pair,^1^ (ii) aqueous biphasic systems induced by the addition of kosmotropic inorganic salts (“salting-out”),^2^ and (iii) thermally responsive systems displaying upper (UCST) or lower (LCST) critical solution temperatures.^3,4^

UCST behavior, where miscibility increases with increasing temperature, is generally consistent with conventional expectations of entropy-driven mixing. More unusual is lower critical solution temperature (LCST) behavior, in which ILs become less miscible with water as temperature increases. LCST phenomena are more commonly associated with polymer solutions, the canonical example being poly­(*N*-isopropylacrylamide) (PNIPAM),^5,6^ and have recently been reported for a range of ionic liquids^7–14^ with potential applications highlighted for refrigeration, dehumidification, desalination, and water harvesting.

LCST behavior requires a subtle balance of thermodynamic effects: a weakly negative enthalpy of mixing that favors miscibility at low temperature, combined with an overall negative entropy of mixing that drives phase separation upon heating. Since the change in Gibbs free energy (Δ*G*) for mixing is negative below the LCST and positive above, consequently, the bulk change in entropy (Δ*S* = −(dΔ*G*/d*T*)) for mixing must also be negative. This contrasts with the more usual, and intuitive case of a positive entropy gain upon mixing. This counterintuitive entropy change, first formalized by Hirschfelder et al.^15^ implies a temperature-dependent restructuring of directional interactions such as hydrogen bonding within the liquid. The LCST behavior of PNIPAM polymer–water mixtures^5,6^ is ascribed to a transformation of the polymer chains from extended chain to globular arrangements in solution and has been rationalized in terms of this structural reorganization excluding water leading to an unusual negative entropy of mixing.^16^ The behavior of some amino acid-based ILs in water^17,18^ has also been explained in a similar manner, in terms of anion–anion hydrogen bond-driven aggregation reminiscent of protein folding; however, these models do not transfer directly to an understanding of the mechanism of LCST behavior in simple ionic liquid-water mixtures containing only single pairs of cations and anions.

ILs that exhibited LCST behavior with water were first identified by Ohno and co-workers^3,4^ who first described these characteristics for a range of IL/water systems most prominently with [P_4444_]^+^ cations paired with either aromatic sulfonate anions (tosylate, 2,4-dimethylbenzenesulfonate, 2,4,6-trimethylbenzenesulfonate) or trifluoroacetate. Ando et al.^19^ subsequently showed that the corresponding salicylate and terephthalate salts also exhibited LCST behavior, and Moura et al.^20^ later extended this to the bioisosteric phenyltetrazolate anions, suggesting that an amphilphilic anion motif helps facilitate this behavior.

LCST behavior is not limited to salts with [P_4444_]^+^ cations, although this cation does appear to be near the optimal balance between hydrophilicity and hydrophobicity. With tetrapentylphosphonium cations, the same anions described above give rise to permanent biphasic systems and to completely water-miscible ILs with tetrabutylammonium cations, except for 2,4,6-trimethylbenzenesulfonate, the most hydrophobic of the benzenesulfonate anions examined, which shows LCST behavior. Similarly, Kohno and Ohno^4^ also showed that tributyloctylphosphonium bromide exhibited LCST behavior in water, whereas [P_4444_]Br does not.

Increasing anion hydrophobicity by, for example, increasing the hydrocarbon or fluorocarbon content in the anion also results in a lowering of the LCST (e.g., from ca. 55 °C ([P_4444_]­[OTs])^21^ to ca. 35 °C ([P_4444_]­[DMBS])^8,21^ to ca. 30 °C ([P_4444_]­[TMBS])).^8,21^ LCST behavior can be lost completely when more hydrophilic anions are used, for example, when [P_4444_]^+^ is paired with benzenesulfonate (cf. tosylate, 2,4-dimethylbenzenesulfonate, 2,4,6-trimethylbenzenesulfonate) or acetate (cf. trifluoroacetate). The complete miscibility of [P_4444_]­[Bz] with water can be contrasted with LCST-phase behavior of [P_4444_] salicylate and terephthalate, despite these latter two systems having anions containing an additional hydrophilic functional group,^19^ and demonstrates that considering apparent hydrophobicity of a single ion in isolation can be misleading. This is especially critical, as the transition from a fully mixed single phase through to a biphasic mixture on heating through the LCST point must lead to changes in both ion–water and ion–ion association and raises the question of how differences in anion–water interactions contribute to the entropy-dominated driving forces behind LCST-phase splitting in IL/water mixtures.

With the thermodynamic description for LCST behavior requiring an overall negative change in entropy on demixing with increasing temperature (and so system enthalpy), it is not directly obvious how the IL-water mixtures undergo such a transition. A mechanistic picture has been proposed, from dynamic light scattering method and molecular dynamics simulations, for aqueous tetraalkylphosphonium benzenesulfonates in which heating reduces ion solvation, shifting the equilibrium from water-separated ion-pairs toward increased cation–anion contact and formation of an IL-rich “water-in-IL” environment, while simultaneously generating a water-rich solvated-ion second phase through condensation of IL-aggregates.^22^ However, the experimental data does not conclusively show whether the results represent equilibrium dispersions of components across the two phases, or the presence of noncoalesced droplets formed above the cloud-point.

The absence of a rationalization of the overall positive Gibbs free energy (Δ_mix_ *G*) of mixing that is a consequence of demixing has not been addressed and the need for molecular-level detail has motivated this study of the thermodynamics of hydration and solution using Isothermal Titration Calorimetry (ITC) and neutron scattering to examine the relative magnitude and enthalpic origins of LCST behavior in tetrabutylphosphonium 5-phenyltetrazolate/water ([P_4444_]­[Ph–tet]/H_2_O)^20^ in contrast to the absence of this behavior in the structurally related, but fully water-miscible analogue, tetrabutylphosphonium benzoate/water ([P_4444_]­[Bz]/H_2_O)^3^ (Figure 1) through measurement of their enthalpy of solvation. Further insight into the mechanism of the phase transition is obtained from a neutron diffraction and isotopic substitution (NDIS) studies. We have examined [P_4444_]­[Ph–tet]/H_2_O mixtures (5.6 waters per IL), corresponding to ca. 20 wt % water which remains in the continuous IL-rich composition region of the phase diagram to evaluate the water and ion–ion environments in the IL-rich phase region and unifies macroscopic thermodynamic evidence with atomic-level structural validation.

**Figure 1:**
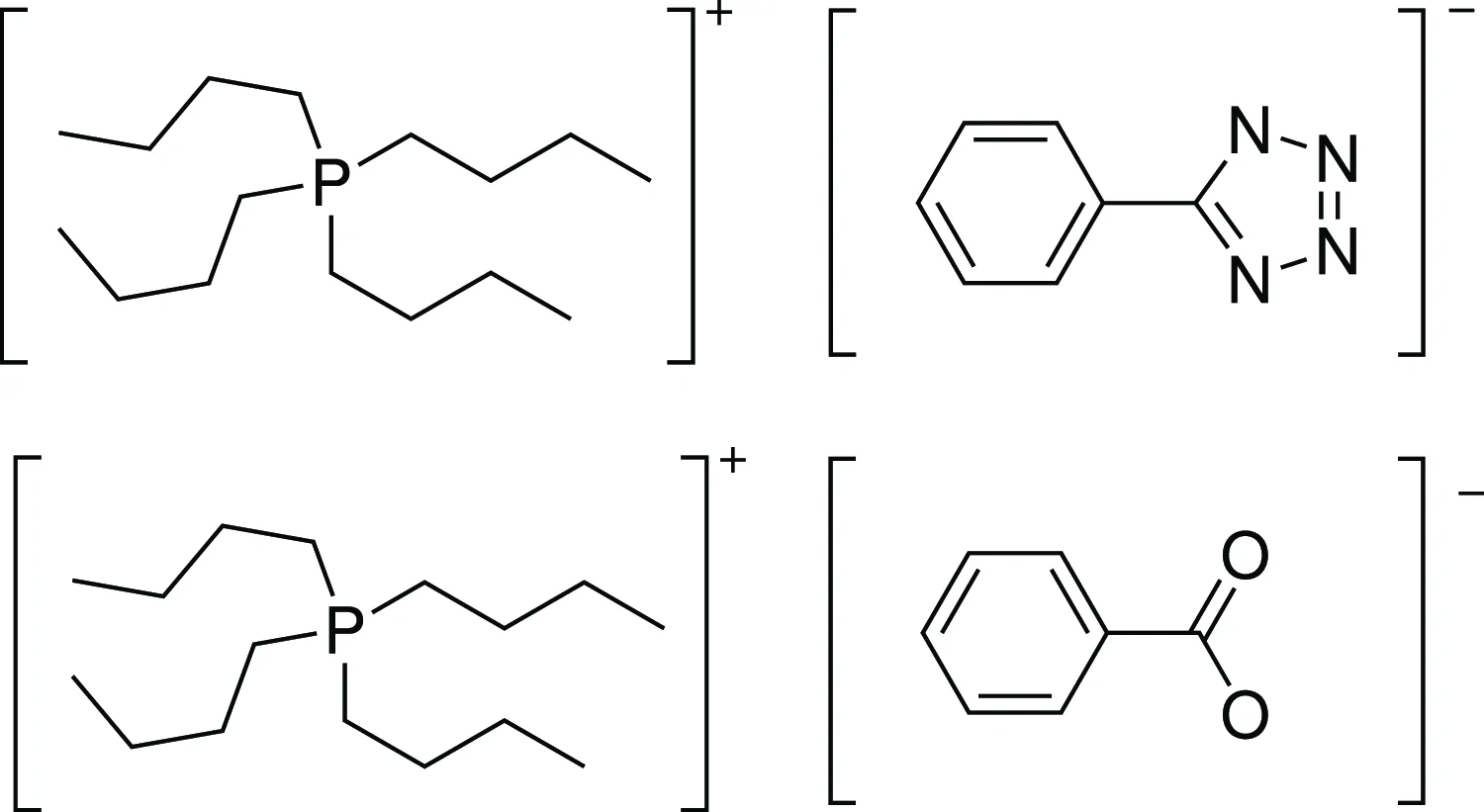
Structures of the two ionic liquids, tetrabutylphosphonium 5-phenyltetrazolate, [P_4444_]­[Ph–tet] (top) and tetrabutylphosphonium benzoate, [P_4444_]­[Bz] (bottom).

## Experimental Section

### Materials

All protiated chemicals including tetrabutylphosphonium hydroxide (40 wt % in H_2_O), 5-phenyl-1*H*-tetrazole, benzoic acid, and D_2_O were purchased from SigmaAldrich. Deuteriated 5-phenyltetrazole-*d*_6_ (>97% purity with deuteriation level determined by GC/MS) was provided by the ISIS deuteration laboratory (preparation is described in SI). The ionic liquids [P_4444_]­[Ph–tet] and [P_4444_]­[Bz] were synthesized by acid–base neutralization of tetrabutylphosphonium hydroxide (40 wt % in H_2_O) with the respective acid, 5-phenyltetrazole or benzoic acid, followed by the removal of water under reduced pressure to yield solid, anhydrous salts. Details are provided in SI, formation of the two ILs were confirmed via ^1^H NMR spectroscopy, mass spectrometry, and melting points which were consistent with literature.^19,20^

### Isotopomer Sample Preparation

Six isotopomeric [P_4444_]­[Ph–tet]/water samples at a 1:5.6 IL:water molar ratio were prepared using protiated tetrabutylphosphonium hydroxide, either protiated or deuteriated 5-phenyltetrazole, and H_2_O or D_2_O as appropriate, with the H/D-distributions between water and the [Ph–tet]^−^ anion shown in Table 1. Samples were prepared by mixing appropriate quantities of the corresponding anhydrous crystalline [P_4444_]­[Ph–tet] salts with either protiated or deuteriated anions with water (H_2_O, D_2_O, or mixtures) at a 1:5.6 molar ratio (80 wt %, χ_IL_ = 0.1510 for the fully protiated sample), followed by stirring at room temperature until homogeneous solutions were obtained.

**Table 1:** Isotopomers, Experimental Densities (AntonPaar DMA 4500M Density Meter, 25 °C), and Corresponding Atomic Number Densities of the [P_4444_]­[Pt-tet]/Water Liquid Samples Investigated with Isotope Substitution on Anion and Water Components with a Common Protiated Cation, [P_4444_]^+^

sample	cation	anion	water	density/g cm^–3^	number density/atoms Å^–3^
1	H	H	H	1.0108	0.09910
2	H	H	H/D		0.09934
3	H	H	D	1.0332	0.09944
4	H	D	H	1.0198	0.09896
5	H	D	H/D		0.09923
6	H	D	D	1.0426	0.10325

### Solid–Liquid Equilibrium Data

The enthalpy of fusion (Δ_fus_ *H*) for [P_4444_]­[Ph–tet] was taken from the literature,^20^ and determined for [P_4444_]­[Bz] using a PerkinElmer Diamond DSC. The DSC was calibrated to the melting point of fusion of water, indium, and lead at 2, 5, 10, and 20 °C min^–1^. The calibration was verified with the melting point of naphthalene and the fusion enthalpy of indium at 5 °C min^–1^. Aluminum pans were hermetically sealed and weighed with a microbalance (resolution of 1 μg). Peaks were carefully integrated at the various scan rates (2, 5, 10, and 20 °C min^–1^) and the final fusion enthalpy was taken as the average of all measured values. The liquidus line for [P_4444_]­[Bz]/H_2_O mixtures was determined using conventional and modulated DSC experiments on a TA Instruments Q2000 DSC equipped with a RSC 90 refrigerated cooling system under nitrogen flow (50 mL min^–1^), along with a temperature precision and accuracy of ±0.01 and ±0.1 °C respectively. On a conventional DSC setup, a heating rate of 2 °C min^–1^, while for a modulated DSC (MDSC) experiment, a heating rate of 3 °C min^–1^, with temperature modulations of ±1 °C was applied for every 60 s.

### Isothermal Titration Calorimetry

Calorimetry measurements for mixing of water into IL/water and IL/water into water were performed using an isothermal titration nanocalorimeter (TA Instruments) housed in a Thermal Activity Monitor (TAM) III thermostat at 25 °C with the temperature maintained within 1 mK. The composition of the mixtures studied is reported in Table 2. The instrument was operated in dynamic heat flow mode at atmospheric pressure. A detailed description of the methodology is reported by Podgoršek et al.^23^

**Table 2:** Samples Prepared for the ITC Experiments^a^

*x*_IL,cell_	*x*_IL,syr_	*w*_IL,cell_	*w*_IL,syr_
[P_4444_][Ph–tet]/H_2_O			
0.090880	0	0.6918	0
0.352125	0	0.9243	0
0.200173	0	0.8490	0
0.358533	0	0.9262	0
0.164328	0	0.8154	0
[P_4444_][Bz]/H_2_O			
0	0.0451	0	0.4991
0.0995	0	0.7000	0
0.1519	0.0455	0.7909	0.5015
0.3275	0	0.9114	0

a *x*_IL,cell_ and *x*_IL,syr_ are the initial mole fraction, and *w*_IL,cell_ and *w*_IL,syr_ are the mass ratio compositions of ionic liquid in the cell and in the syringe, respectively.

A full gain and a dynamic calibration were performed before each experiment. Titration experiments were performed adding small volumes (1–5 μL) of pure water or solutions of IL in H_2_O into the 1 mL stainless steel sample cell containing the same IL/H_2_O solution.

Injections were automated using a motor-driven 3810 syringe pump (TA Instruments) fitted with a gastight Hamilton syringe, with an interval of 120–150 min between injections, to allow the baseline to reestablish. The sample cell was stirred at 60 rpm by using a gold-coated propeller. To ensure a stable baseline, the system was equilibrated both between calibration and injection and between individual injections. Each injection recorded a peak corresponding to the heat effect of the mixing process. Table 2 shows the concentrations prepared for the sample cell and the syringe (or dispenser) during the ITC experiments.

An example of a typical isothermal titration calorimetric enthalpy change profile measured on a single injection is shown in Figure 2 where details of the baseline correction is included. Integration of the area of the peaks from the experimental ITC data allows the determination of *Q_i_*.

**Figure 2:**
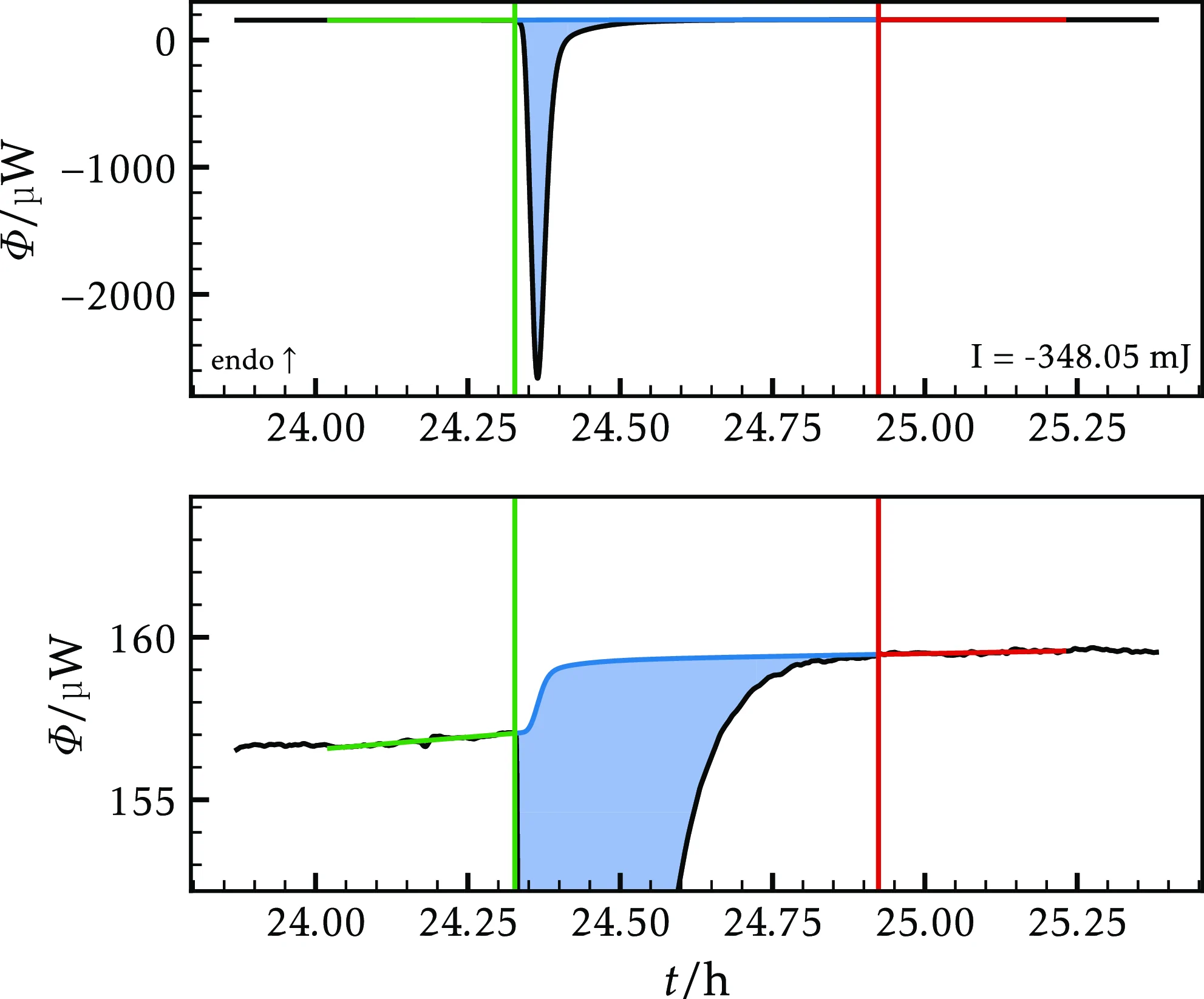
Example of a typical ITC enthalpy change profile on a single injection. The area under the peak (in blue) recorded during the injection is integrated to obtain the value of *Q* (in mJ). In the figure, *I* is the integrated value (=*Q*).

### Dissolution Calorimetry

Because pure [P_4444_]­[Ph–tet] and [P_4444_]­[Bz] are solids at 25 °C, it was necessary to first calculate their dissolution enthalpy (Δ_sol_ *H*) which is the heat change when a small amount of solute (in this case the IL) is dissolved in a solvent (H_2_O).

The heats of dissolution, *Q*_sol_, of [P_4444_]­[Ph–tet] and [P_4444_]­[Bz] in water were measured at 25 °C using an isothermal dissolution calorimeter (IDC) from TA Instruments housed in a TAM IV thermostat operated in a dynamic heat flow mode at atmospheric pressure. A full gain and a dynamic calibration was performed before each experiment. Small quantities (between 6 and 22 mg) of the pure salts were added to an accurately know quantity of pure water contained in a 20 mL stainless steel cell. The sample cell was stirred at 60 or 80 rpm using a gold-coated stirrer. The system was equilibrated before each injection as well as before each two experiments. An empty cartridge was opened in the liquid sample and used as a blank, while the reference cell was filled with an equivalent amount of pure water. Each dissolution recorded a peak corresponding to the heat effect of the dissolving process. Integration of the area of the peak allows determination of *Q*_sol_. Experimental values of *Q_i_* and *Q*_sol_ are presented in the SI.

### Calculation of Excess Properties

To understand the thermodynamics of the complete system of IL/H_2_O mixtures, it is necessary to have information on the total Gibbs free energy of the system, as

1 $$\Delta_{\mathrm{mix}}G=\Delta_{\mathrm{mix}}H-T\Delta_{\mathrm{mix}}S$$

that constitutes both the enthalpic and the entropic contributions to mixing. This information was obtained as excess Gibbs free energy (*G* ^E^) by plotting the phase diagrams of the two IL/H_2_O mixtures.

In an ideal mixture, the phase diagrams are obtained from the properties of fusion of the pure components making up the mixture and is given by eq 2

2 $$T={\left[\frac{1}{T_{\mathrm{fus},i}}-\frac{R\ln x_{i}}{\Delta_{\mathrm{fus}}H_{i}\left(T\right)}\right]}^{-1}$$

where *T*_fus,*i*_ is the fusion temperature of the pure component *i*, *x*_*i*_ is the mole fraction of component *i*, Δ_fus_ *H*_*i*_(*T*) is the fusion enthalpy of component *i* at temperature *T*.

However, for a real mixture, the equation of the phase diagram requires an activity coefficient γ_*i*_ for component *i* to be included as in eq 3, from which the excess Gibbs energy of mixing can then be calculated from the value of γ_*i*_.

3 $$T={\left[\frac{1}{T_{\mathrm{fus},i}}-\frac{R\ln\gamma_{i}x_{i}}{\Delta_{\mathrm{fus}}H_{i}\left(T\right)}\right]}^{-1}$$

Experimentally obtained heats of mixing, *Q_i_*, and dissolution, *Q*_sol_, are used to calculate the partial molar excess enthalpies of the components in the mixture, following the relationship

4 $$\delta Q=\delta n_{1}\left[{\mathrm{H̅}}_{1}^{E}\left(x_{\mathrm{cell}}^{\mathrm{fin}},T\right)-{\mathrm{H̅}}_{1}^{E}\left(x_{\mathrm{syr}},T\right)\right]+\delta n_{2}\left[{\mathrm{H̅}}_{2}^{E}\left(x_{\mathrm{cell}}^{\mathrm{fin}},T\right)-{\mathrm{H̅}}_{2}^{E}\left(x_{\mathrm{syr}},T\right)\right]+n_{1}\left[{\mathrm{H̅}}_{1}^{E}\left(x_{\mathrm{cell}}^{\mathrm{fin}},T\right)-{\mathrm{H̅}}_{1}^{E}\left(x_{\mathrm{cell}}^{\mathrm{ini}},T\right)\right]+n_{2}\left[{\mathrm{H̅}}_{2}^{E}\left(x_{\mathrm{cell}}^{\mathrm{fin}},T\right)-{\mathrm{H̅}}_{2}^{E}\left(x_{\mathrm{cell}}^{\mathrm{ini}},T\right)\right]$$

where, a quantity δ*n*_inj_ = δ*n*_1_ + δ*n*_2_ of a mixture at composition *x*_syr_, is injected into a quantity *n*_cell_ = *n*_1_ + *n*_2_ of another mixture at composition *x*_cell_. Subscripts *i*, cell, and syr refers to component *i*, sample cell and syringe respectively. Superscripts ini and fin refer to before and after injection in the mixtures.^24^

However, when a pure liquid (**1**) is injected into a mixture, δ*n*_2_ = 0 and ${\mathrm{H̅}}_{1}^{l}\left(x_{syr}\right)={\mathrm{H̅}}_{1}^{l,*}\left(T\right)$ , where ${\mathrm{H̅}}_{1}^{l,*}\left(T\right)$ is the partial molar enthalpy of the pure liquid **1**. The partial molar excess enthalpy of the component **1** in the mixture is then calculated as in eq 5

5 $${\mathrm{H̅}}_{1}^{E}=\frac{\delta Q_{i}}{\delta n_{1}}$$

In the same manner, during the dissolution experiments when δ*n*_1_ = 0, from the heat of dissolution (*Q*_sol_), the partial molar excess enthalpy of component 2 (*H̅*_2_^E^) was calculated by correcting it from the heat of melting or enthalpy of fusion (Δ_fus_ *H*) of component 2, following eq 6

6 $${\mathrm{H̅}}_{2}^{E}=\frac{\delta Q_{sol}}{\delta n_{2}}-\Delta_{\mathrm{fus}}H_{2}\left(T\right)$$

Finally, to integrate this data (partial molar excess enthalpies) and obtain the total enthalpy of mixing (*H* ^E^ or Δ_mix_ *H*), the *H̅*_1_^E^ from ITC and *H̅*_2_^E^ from dissolution calorimetry were fitted to Legendre polynomials^25^ using eq 4. To prevent the optimization process from being disproportionately influenced by variations in injection size, the measured heat effects were normalized by dividing by the total injected quantity (δ*n*_1_ + δ*n*_2_) as δ*Q*/(δ*n*_1_ + δ*n*_2_). This normalization ensures that contributions from larger injections do not dominate the fitting process, thereby improving the accuracy and stability of the resulting model. Thus

7 $$\Delta_{\mathrm{mix}}H\left(x\right)=x\left(1-x\right)\sum_{i=0}^{n}a_{i}L_{i}\left(1-2x\right)$$

where *n* is the degree of the fitting polynomial, {*a*_*i*_}_*i*∈[[0,*n*]]_ are the fitting parameters, and *L*_*i*_ is the Legendre polynomial of degree *i*.

### Neutron Scattering

Neutron diffraction experiments were performed on NIMROD (ISIS Pulsed Neutron and Muon Source, Rutherford Appleton Laboratory) over a *Q* range from 0.02 to 50 Å^–1^ (with *Q* = 4π sin θ/λ, where 2θ = scattering angle and λ = wavelength). Data were collected for the six [P_4444_]­[Ph–tet]/water (χ_IL_ = 0.1510, 1:5.6 IL:water) monophasic composition shown in Table 1, at 25 °C in “null scattering” Ti_0.68_Zr_0.32_ flat plate cells with internal geometries of 1 mm × 35 mm × 35 mm and a wall thickness of 1 mm. The temperature maintained using a recirculating heater (Julabo FP50).

Experimental sample densities for samples **1**, **3**, **4**, and **6** were measured at 25 °C. Atomic densities for the mixed compositions (**2** and **5** containing H/D-water) were taken as the median values between the corresponding protiated and deuterated variants. The corresponding scattering levels obtained were consistent with the isotopic compositions of the samples. Corresponding atomic number densities (atoms Å^–3^) were calculated within GUDRUN from the density and atomic composition as indicated in Table 1, and the averaged number density (0.100 ± 0.018 atoms Å^–3^) was used to construct and fill the simulation box for Empirical Potential Structure Refinement (EPSR)^26,27^ modeling. Absolute values of the differential cross sections were obtained from the raw scattering data by normalizing against a 3.1 mm thick null-scattering vanadium–niobium alloy (0.9485:0.0515) standard and correcting for background and multiple scattering, container scattering and self-attenuation, using GUDRUN.^28^ The neutron scattering data obtained from GUDRUN was analyzed using EPSR^26,27^ to fit a self-consistent atomistic model of the liquid correlations.

The EPSR approach consists of a Monte Carlo simulation, using Lennard-Jones potentials with atom-centered point charges that are combined with basic information about the structure of the atoms or molecules present in the system and total atomic densities of the system to constrain the model in a chemically and physically reasonable manner. By comparison of the differences between calculated and experimental structure factors in *Q*-space for each data set, an empirical perturbation potential is determined. This is combined with the reference potential and used as the new potential for simulations, iteratively driving the model toward agreement with experimental data.

EPSR of the [P_4444_]­[Ph–tet]/H_2_O model was performed against the experimental data for the six isotopologues simultaneously over the range (*Q* = 0.1–30 Å^–1^), truncating the low *Q* (small angle) data accessible with NIMROD as no evidence for long-range aggregation was evident. Refinement was initialized using an equilibrated Monte Carlo simulation containing 760 discrete species (560 H_2_O molecules, 100 [P_4444_]^+^ cations and 100 [Ph–tet]^−^ anions) in a cubic box with sides 47.17 Å consistent with the experimental density, and was equilibrated for over ca. 10k cycles before accumulating and averaging structural data. Initial interatomic distance constraints used to define the basic molecular geometries for the [P_4444_]^+^ cation and [Ph–tet]^−^ anion were generated using the LigParGen OPLS/CM1A parameter generator^29–31^ and the geometries compared to those from the X-ray crystal structure of [P_4444_]­[Ph–tet].^20^ For water, SPC^32^ nonbonded parameters were used. Atom types are defined based on unique positions in the molecular skeletons (Figure 3). Full parameters of the reference potential and interatomic distance and angular constraints used to define the basic molecular geometries are given in Tables 3 and 4.

**Table 3:** Lennard-Jones Well Depth (ϵ), Range (σ), and Charge (*q*) Parameters Used for the Reference Potential of the EPSR Model

atom label	ϵ/kJ mol^–1^	σ/Å	*q*/e
Cation			
P1	0.83680	3.74	2.35250
C1	0.27614	3.50	–0.74605
H1	0.12552	2.50	0.10115
HM	0.12552	2.50	0.10115
C2	0.27614	3.50	–0.14394
CM	0.27614	3.50	–0.21458
Anion			
CA1	0.29288	3.55	0.0677
CA2	0.29288	3.55	–0.1222
CA3	0.29288	3.55	–0.1485
CA4	0.29288	3.55	–0.1665
CAN	0.43932	3.55	–0.0415
NA1	0.71128	3.25	–0.3483
NA2	0.71128	3.25	–0.1609
HA	0.12552	2.42	0.1234
Water			
OW	0.63640	3.1506	–0.66720
HW	0.00000	0.0000	0.33360

**Table 4:** Intramolecular Bond Distance and Angle Constraints Used to Define the Basic Geometries of [P_4444_]^+^, [Ph–tet]^−^, and H_2_O in the Initial EPSR Simulation Model

bond distance/Å		bond angle/°	
Cation			
P–C	1.838	all cation bond angles 109.5	
C–C	1.527		
C–H	1.093		
Anion			
CA–CA	1.42	CA–CA–H	119.84
CA–H	1.10	CA–CA–CA	120.09
CA–CA	1.39	CA–CAN–NA1	126.62
CAN–NA1	1.41	CA–CA–CAN	120.67
NA1–NA2	1.29	CAN–NA1–NA2	105.52
NA2–NA2	1.33	NA1–CAN–NA1	106.77
		NA1–NA2–NA2	111.09
Water			
OW–HW	0.96	HW–OW–HW	103.51

**Figure 3:**
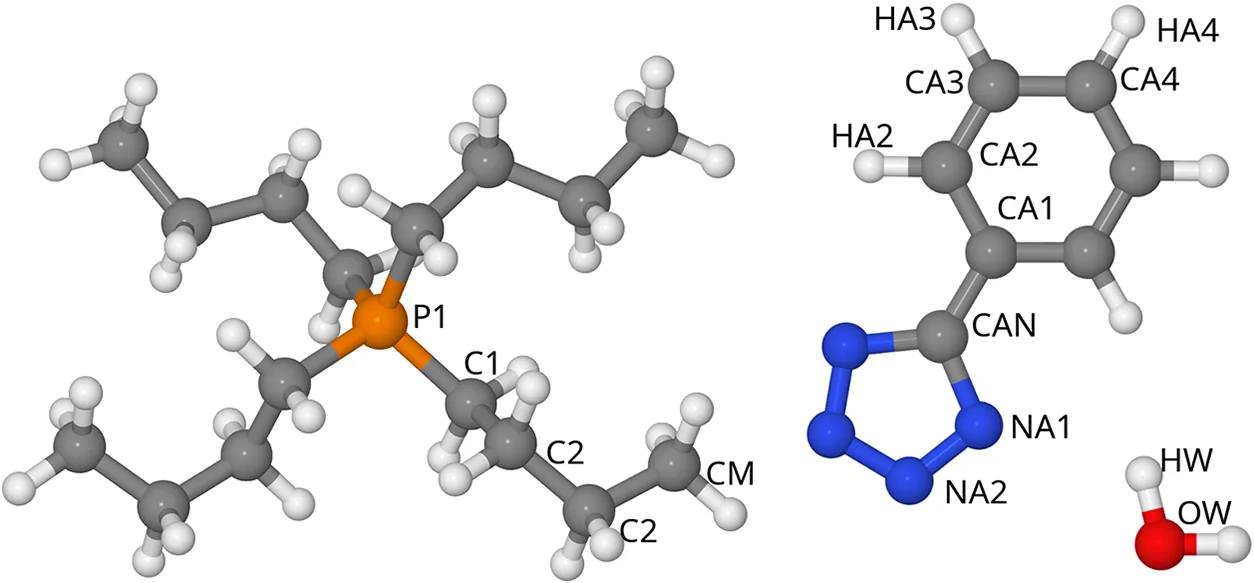
Atom labels for specific atomic sites in [P_4444_]^+^ cation, [Ph–tet]^−^ anion, and H_2_O molecules used in the EPSR simulation model.

## Results and Discussion

The objective of these studies is to try to elucidate information regarding the driving forces behind the thermally induced phase splitting of [P_4444_]­[Ph–tet]/water mixtures^20^ and other related IL/water systems with LCST behavior;^3,4,8–14^ in particular, to determine whether the balance of enthalpy/entropy of mixing/demixing can be determined and understood within the context of potential changes in the ion–ion and ion–water interactions present in the solutions that could guide the rational design of new fluids with LCST behavior.

### Enthalpies of Mixing from ITC

The mixing enthalpies of the two ILs, [P_4444_]­[Ph–tet] and [P_4444_]­[Bz], with water were calculated from the isothermal titration and dissolution calorimetry data (SI) and the results are shown in Figure 4.

**Figure 4:**
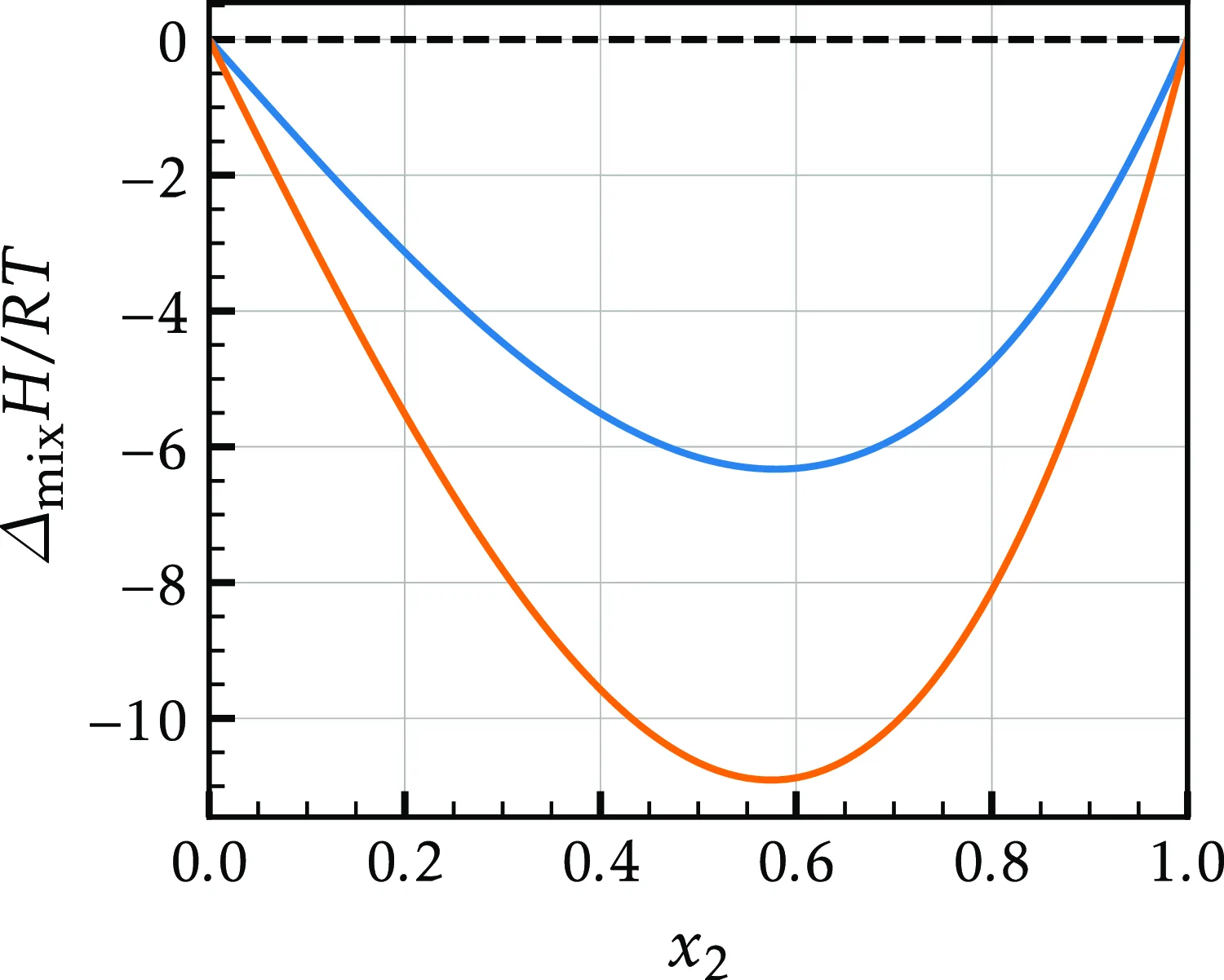
Mixing enthalpies at 298 K for [P_4444_]­[Ph–tet] (blue line) and [P_4444_]­[Bz] (orange line) with water obtained from isothermal titration calorimetry data and fit of partial molar excess enthalpy to Legendre polynomials.

The mixing enthalpies show minima at compositions around mole fraction IL, *x*_IL_, of ≈0.55–0.6. However, the minimum of the enthalpy for [P_4444_]­[Bz]/H_2_O is approximately twice as negative as that for [P_4444_]­[Ph–tet]/H_2_O, indicating more favorable IL/water interactions in the benzoate salt. This observation is consistent with the broader miscibility window of [P_4444_]­[Bz] in water near room temperature, assuming an ideal liquidus line at ionic liquid mole fractions above 0.5.

Interestingly, mixtures of H_2_O with [P_4444_]­[Ph–tet] exhibit nearly ideal behavior over a broad composition range (*x*_2_ ≥ 0.35). At lower mole fractions (*x*_2_ ≤ 0.35), however, the system displays more complex phase behavior as previously described in Moura et al.^20^ including two eutectic points and significant deviations from ideality. Combined with the measured negative enthalpy of mixing, this implies a negative entropy of mixing, which accounts for the observed demixing upon increasing temperature.

This behavior contrasts with that of [P_4444_]­[Bz] with H_2_O across the same composition range, where a pronounced depression of the melting temperature is observed. At higher water contents, the [P_4444_]­[Bz]/H_2_O system exhibits increasingly complex thermal features, as evidenced by differential scanning calorimetry (DSC) measurements (see SI), which reveal multiple endothermic transitions contributing to several apparent melting points in the phase diagram (Figure 5b). Such behavior suggests the presence of multiple solid phases that are eventually metastable states, and we attribute the two transitions to the formation of hydrate-like structures, where water molecules organize into cage-like, “clathrate-hydrate” arrangements that encapsulate the ionic liquid species.^33–35^ In this scenario, ice crystallization occurs in a structured manner around [P_4444_]­[Bz], leading to distinct thermal events eventually associated with the melting of these different supramolecular assemblies.

**Figure 5:**
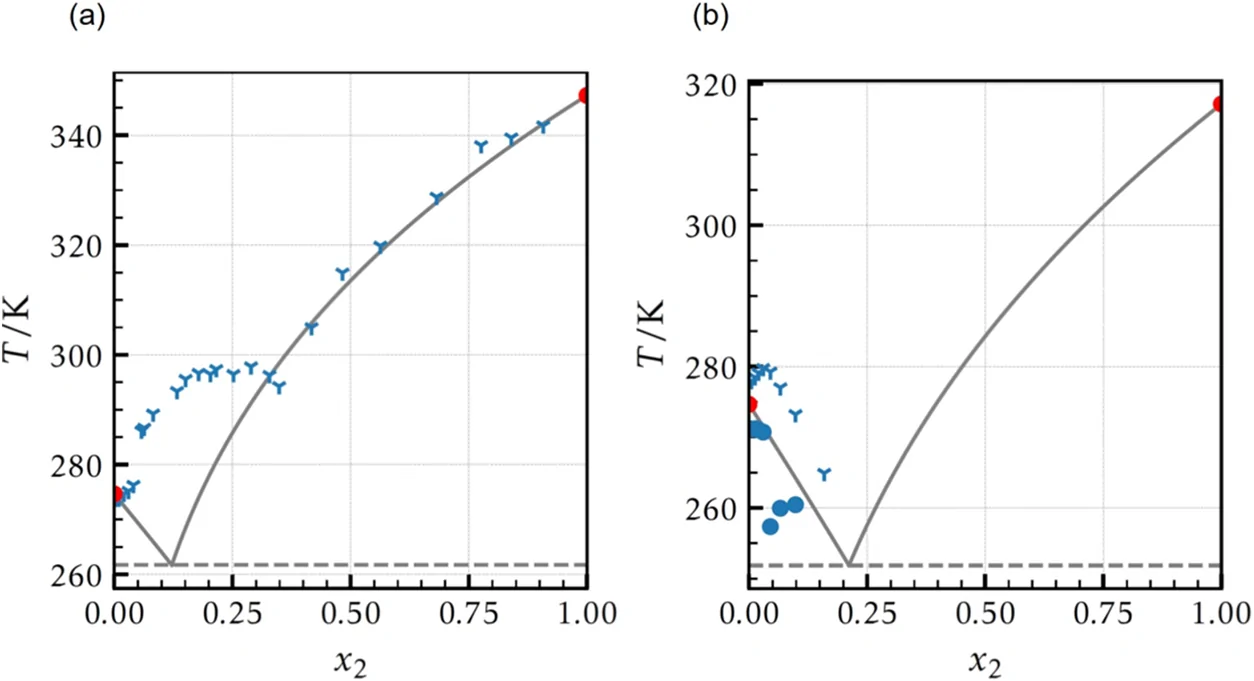
Phase diagram of the mixtures: (a) [P_4444_]­[Ph–tet]/H_2_O^20^ and (b) [P_4444_]­[Bz]/H_2_O. Blue symbols are experimental data points and gray solid line is the ideal phase diagram. In the case of [P_4444_]­[Bz]: Y corresponds to the inflection points of the last (higher temperature) DSC peak; circles represent the temperatures of the onset of the first (lower temperature) DSC peak (SLE data for [P_4444_]­[Ph–tet]/H_2_O adapted from Moura et al.^20^ and for [P_4444_]­[Bz]/H_2_O see Supporting Information).

### Neutron Scattering and Molecular-Scale Structure

For all of the experimental samples, the cation was protiated. Efforts to synthesize perdeuterated [P_4444_]^+^ cations, either by deuteration of tributylphosphine and subsequent alkylation following the method described for the preparation of perdeuterated trihexyltetradecylphosphonium cations,^36,37^ or by direct catalytic deuteration of tetrabutylphosphonium salts with D_2_O over Pt/C,^38^ were unsuccessful at scale (see SI).

The data from [P_4444_]­[Ph–tet]·5.6H_2_O (samples **1**–**6**) was modeled using the descriptors for the species present (cation/anion/water) shown in Tables 3 and 4. Despite the large protiated component in all of the samples, with a maximum 1:2.2 D:H ratio in the most deuterium-rich sample (**6**) as a consequence of lack of availability of perdeuterated [P_4444_]^+^ cations, inelastic scattering in the raw data were well addressed with the background correction routines in GUDRUN, producing a good, consistent fit between the model and experimental data sets. Comparisons of experimental and simulated total structure factors, *F*(*Q*), and the corresponding Fourier transforms to real space, *G*(*r*), for each of the six isotopically distinct experimental mixtures (Figure 6, *left* and *center*) show the quality of fit to the experimental data.

**Figure 6:**
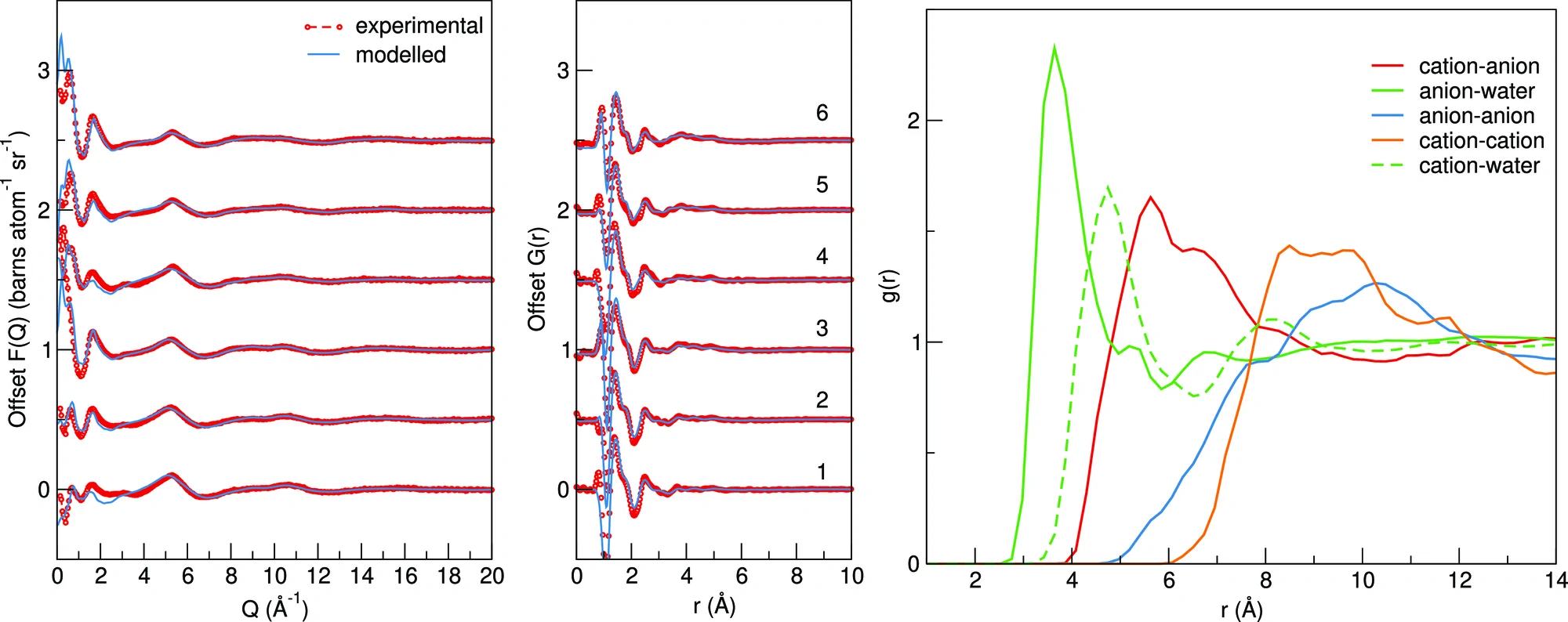
Experimental *F*(*Q*) data (red symbols), EPSR models for the data (blue lines) and residual differences between simulated and experimental data (dashed lines) (*left*), and the Fourier transform to real space *G*(*r*) (*center*) for the six isotopomeric [P_4444_]­[Ph–tet]·5.6H_2_O mixture samples from Table 1, at 25 °C, and center of mass radial distribution functions (*right*) between components extracted from the EPSR models.

Center of mass (COM) radial distribution functions (RDFs) were calculated using the SHARM routines within EPSR, for each of the components in the systems (Figure 6, *right*). The RDFs reveal the anticipated Coulombic arrangement of the IL components, with a broad cation–anion association present with a first shell maximum around 5.7 Å and each ion surrounded, in their second shell, by similar ions (i.e., with cation–cation and anion–anion correlations with a broad maxima around ca. 8–12 Å). The transition from first to second shell correlation is at ca. 8 Å with a large degree of crossover between the broad peaks in the RDFs. These broad, and overlapping, RDFs are consistent with the large volume of the [P_4444_]^+^ cation.

Both cation–water and anion–water RDFs show more defined COM correlations in their first shell and are observed at shorter distances (4.8 and 3.7 Å respectively) than the cation–anion first peak (5.7 Å). Again, this is consistent with the differences in size between the two ions and water molecules. However, simply considering the COM RDFs can give a distorted view of coordination environments and interactions. This can be particularly evident when components are of dissimilar sizes, or where the interaction sites are significantly displaced from the center of mass preventing ready reduction of the three-dimensional interaction profile into a one-dimensional, isotropic RDF.

The positions of the first maxima (*r*_max_) in the correlations and coordination number (*N*_C_) calculated to the minimum after this peak (*r*_min_) for the first shell site–site partial radial distribution functions (pRDFs) between water (HW and OW) with both [Ph–tet]^−^ anions and [P_4444_]^+^ cations are listed in Table 5 with the water–cation and water–anion pRDFs shown in the SI. As expected, there is little evidence of strong hydration or correlation of water with the cation. The first shell contacts occur at 3.5–2.6 Å between the cations alkyl-chains and water OW sites. It is clear from these pRDFs that the water–cation associations are unstructured with lower peak heights and broad distributions and notably, with an even wider spread of nondirectional correlation with HW sites associated with potential C–H···OW interactions.

**Table 5:** Coordination Numbers within the First Shell Correlations with Water (HW/OW) at 25 °C Showing Position of the First Correlation Shell Maximum (*r*_max_) and the Minimum after This Peak (*r*_min_) to Which the Coordination Numbers (*N*_C_) Were Calculated

central atom	probe	*r*_max_	*r*_min_	*N*_C_	central atom	probe	*r*_max_	*r*_min_	*N*_C_
Water–Anion									
NA1	HW	2.0	2.7	2.0	NA1	OW	2.8	4.0	2.5
NA2		2.0	2.7	2.4	NA2		2.9	4.0	3.1
CAN		2.7	4.0	5.8	CAN		3.3	4.6	3.7
CA1		3.5	5.0	7.4	CA1		4.0	5.5	4.9
CA2		4.3	5.2	6.7	CA2		3.6	4.2	1.3
CA3		4.4	5.0	5.2	CA3		3.6	4.2	1.1
CA4		4.4	5.0	4.8	CA4		3.6	4.2	1.3
Water–Cation									
P1	HW	5.0	6.5	13.1	P1	OW	4.7	6.5	6.5
C1		4.3	5.4	6.2	C1		3.6	4.4	1.6
C2		4.2	5.4	7.4	C2		3.5	4.4	2.0
CM		3.9	5.4	7.7	CM		3.5	4.4	2.4

Selected pRDFs between water (HW and OW) and the [Ph–tet]^−^ anion are plotted in Figure 7 along with the spatial distribution of water–anion correlations showing the positions of highest probability of water–anion contact. The pRDFs indicate the greatest preference for correlation is from HW to the negatively charged tetrazolate group, with slightly shorter *r*_max_ for the two HW–N correlations (2.0 Å) than for HW–CAN (2.7 Å), although as shown in Figure 7, all three correlations contribute to the same first shell peak to the first minimum at ca. 5.0 Å. Notably, the profile of the correlation of water molecules around the tetrazolate ring in Figure 7 shows very little evidence for structured association, i.e., hydrogen bonding from water HW to specific atom sites on the tetrazolate ring. The water HW–tetrazolate ring correlation extends as a radial “doughnut” encompassing all three unique sites (CAN, NA1, and NA2) and the accessible volume above and below the plane of the aromatic ring, as well as a lobe associated with a more defined radial interaction with the two NA2 atom sites. This latter correlation has the largest peak height (greatest order) of the three HW-anion pRDFs in Figure 7. However, strong directional HW-anion hydrogen bonding would be anticipated to manifest in a much greater probability density for interactions within the plane of the tetrazolate ring, rather than above/below the ring. Similarly, between water and the phenyl-group of the anion; the C–H···OW pRDFs that would be anticipated to reveal interactions show a step in probability of contact at ca. 3.0 Å with almost no evidence of structure other than the step in probability starting from the van der Waals separation distance from 2.5 to 2.7 Å.

**Figure 7:**
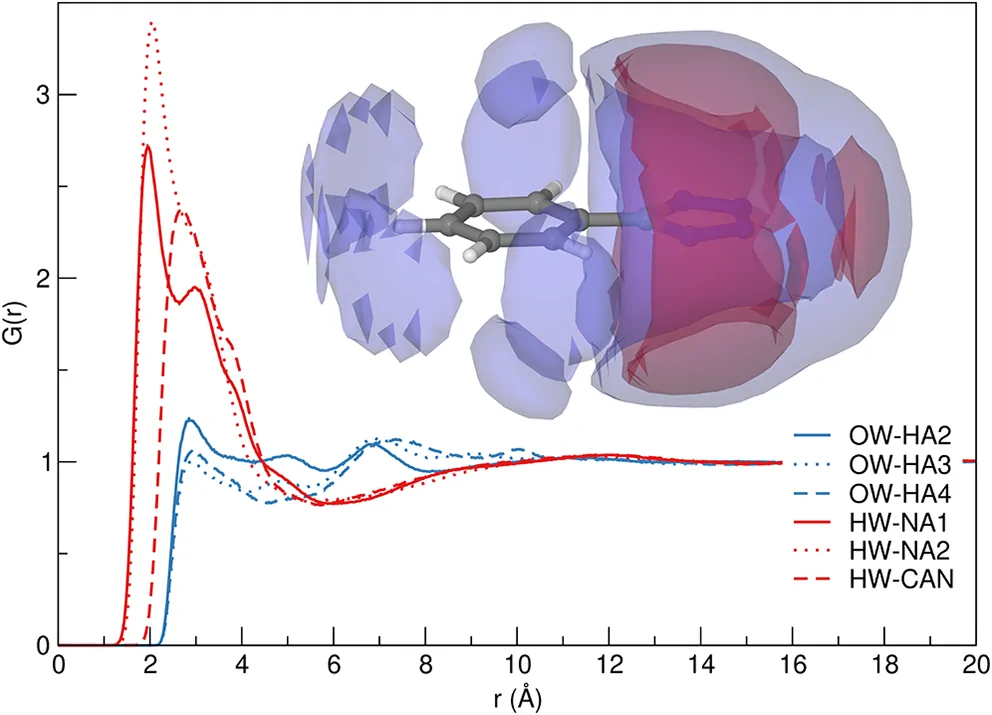
Water–anion site–site partial radial distributions (HW–tetrazolate sites and aromatic H–OW) in the [P_4444_]­[Ph–tet]·5.6H_2_O and the corresponding center of mass spatial distribution of waters around the [Ph–tet]^−^ anion showing the top 25% (red) and 50% (blue) probability distributions within the water–anion first shell to 7 Å.

If focus is turned to the water local environment, water molecules are surrounded by cation and anion, with broad and expected segregation,^39^ i.e., with H-bonding/electrostatic interactions from HW to anion, and from cation to OW as shown in Figure 7. This resolves, in the water centered SDF (Figure 8) to show only a weak water–water correlation and much loser and dominant ion–water association consistent with the correlations cation–OW and anion–HW interactions in the pRDFs.

**Figure 8:**
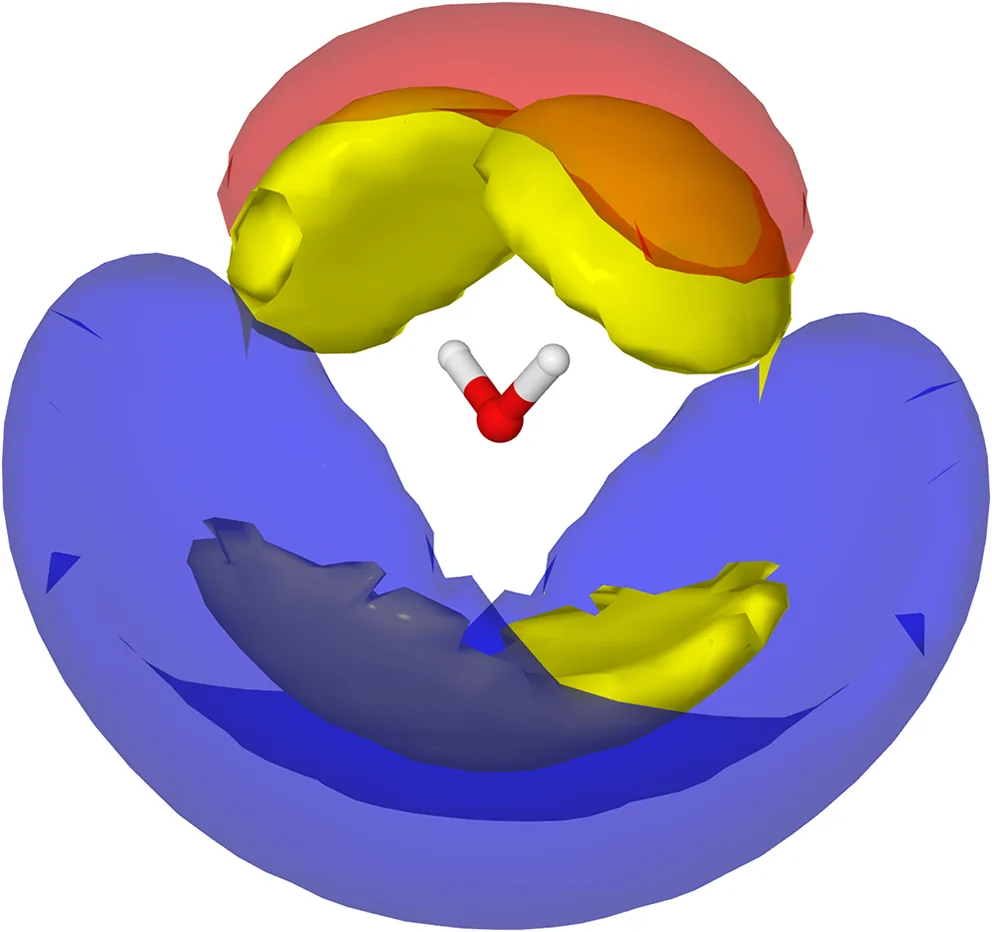
Spatial distributions of water, cations, and anions around a single water molecule as the center of reference showing the top 25% probabilities within the first correlations shells to water (yellow, to 4.5 Å), cations (blue, to 6.5 Å), and anions (red, to 6.0 Å).

Furthermore, performing a water cluster analysis where the probability that water molecules within the EPSR simulation are hydrogen-bonded to other water molecules (with the criteria that pairing in a cluster occurs if HW···OW distances are ≤ 2.5 Å between separate water molecules) reveals (Figure 9) that 77% of water molecules are isolated within the IL with no neighboring water molecules within 2.5 Å. A further 12% are hydrogen-bonded to a single additional water molecule (cluster size = 2), and a further 7% participate in clusters of 3 or 4 water molecules.

**Figure 9:**
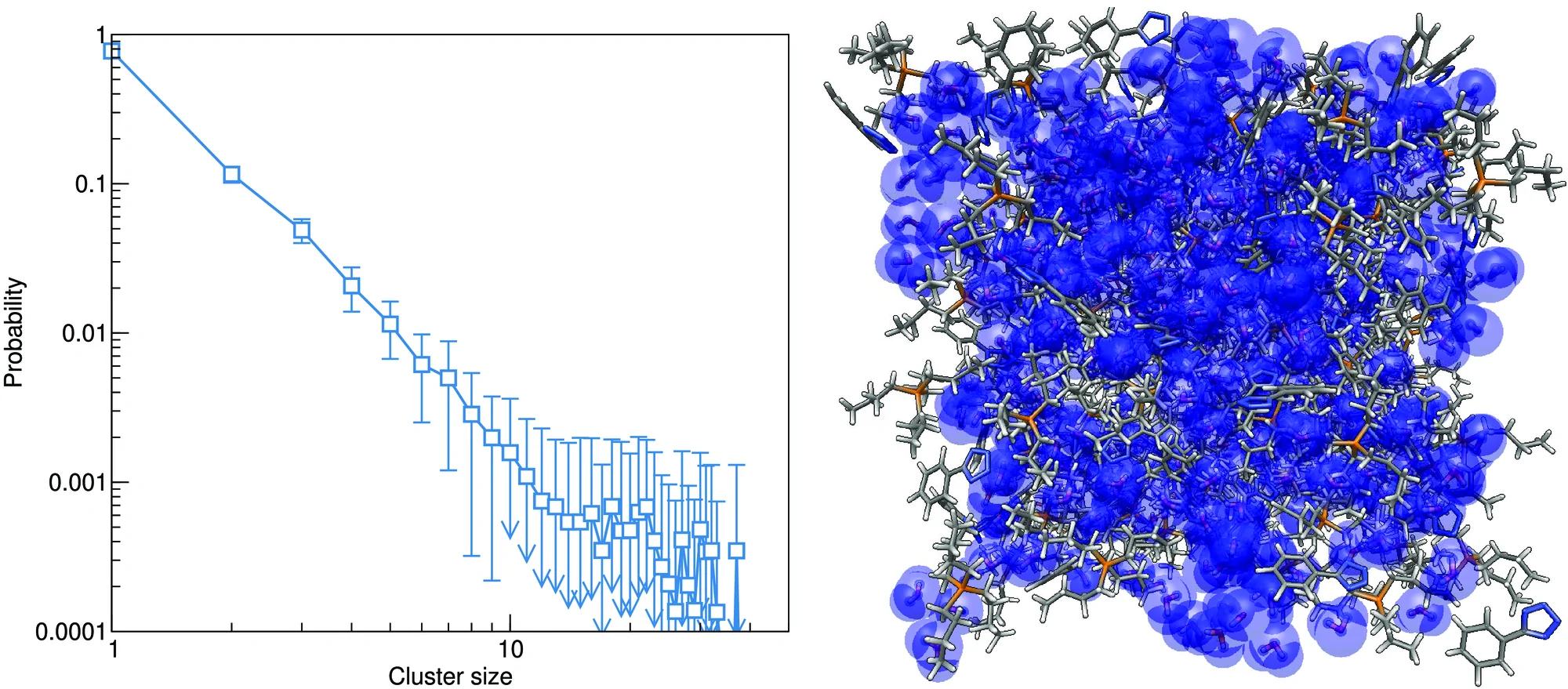
Water–water cluster distributions (*left*) in the EPSR model of [P_4444_]­[Ph–tet]·5.6H_2_O at 25 °C determined with a separations of pairs of HW and OW atoms in individual molecules of ≤2.5 Å to be counted as part of a cluster. This is evidence for lack of a distinct water percolation network, as can be seen in the simulation snapshot (*right*) where the water molecules are highlighted.

This results suggest that [P_4444_]­[Ph–tet]/H_2_O mixtures in the IL-rich side of the phase diagram behave as hydrophobic ILs with the excess water dispersed within the matrix embedded as isolated water molecules^40^ between cation and anion pairs (note this is not to imply that the IL consists of ion-pairs, rather that the water molecules sit within the ionic matrix as discussed by Welton and co-workers^41,42^ ).

### Mechanism of LCST Formation

The combined calorimetric and structural evidence indicates that LCST-phase separation in [P_4444_]­[Ph–tet]/H_2_O arises from an entropically driven loss of anion solvation. In contrast, the benzoate anion forms much stronger hydration shells, leading to more negative mixing enthalpies that suppress LCST behavior almost entirely. Of greatest importance is the relative hydrophobicity of the cation–anion pair in water, which can be quantified through ITC excess enthalpy measurements to extract Δ_mix_ *H*. This change in mixing enthalpy with temperature, Δ_mix_ *H*(*T*_2_,*x*), is linked to the excess heat capacity *C*_p_^E^ according to

8 $$\Delta_{\mathrm{mix}}H\left(T_{2},x\right)=\Delta_{\mathrm{mix}}H\left(T_{1},x\right)+\int_{T_{2}}^{T_{1}}C_{p}^{E}\left(T,x\right)dT$$

From eq 8, with an excess heat capacity of the order of 10 J mol^–1^ K^–1^, over a temperature range of 50 K (which is overestimated for the change required to achieve the LCST transition), the error in calculation of mixing enthalpy can be estimated to be in the order of 0.5 kJ mol^–1^ compared to the difference in mixing enthalpies that are in the order of kJ mol^–1^. Thus, over the small temperature range that is under consideration with respect to LCST behavior, the temperature dependence of *C*_p_^E^ can be neglected.^43^

This estimate is consistent with the magnitudes of data reported for IL + water mixtures. Rebelo et al.^44^ examined the excess properties of 1-butyl-3-methylimidazolium tetrafluoroborate + water, an IL that has an UCST around 280 K. Excess molar heat capacities, *C*_p_^E^, were reported to range from −1.0 to 10 J mol^–1^ K^–1^ depending on composition, with a maximum temperature variation of <0.3 J mol^–1^ K^–1^ between 278.15 and 333.15 K. Even for strongly interacting protic pyrolidinium nitrate + water^45^ with a large *C*_p_ that can be ascribed to extensive hydrogen bonding to and from both ions and water molecules, excess molar heat capacities, *C*_p,max_^E^, ranged from +14 J mol^–1^ K^–1^ for IL-rich compositions to *C*_p,min_^E^ −14 J mol^–1^ K^–1^ in water-rich mixtures. Further measurements of excess heat capacities of IL + water systems have been reviewed^46^ and across the data surveyed, excess heat capacities ranged from *C*_p,max_^E^ 0.4 to 23.7 J mol^–1^ K^–1^ and *C*_p,min_^E^ −0.4 to −14.2 J mol^–1^ K^–1^, typically with S-shaped curves with the average range in Δ*C*_p,min/max_^E^ of 11 J mol^–1^ K^–1^.

Excess enthalpies can provide a starting point for understanding species interactions and reflect the difference in the strength of interactions between unlike species compared to like species, and in water, this reflects the capacity to form hydrogen bonds with water molecules.

The more negative enthalpy of mixing observed for [P_4444_]­[Bz]/H_2_O mixtures compared with [P_4444_]­[Ph–tet]/H_2_O indicates stronger ion solvation. Because both systems contain the same [P_4444_]^+^ cation, this difference must arise predominantly from stronger anion–water interactions. In the [P_4444_]­[Bz]/H_2_O system, the more favorable (more negative) enthalpy of mixing stabilizes a single homogeneous phase, which exhibits the expected “normal” behavior by remaining monophasic upon heating.

The much smaller (less negative) enthalpy of mixing in the [P_4444_]­[Ph–tet]/H_2_O systems reflects weaker hydration of the [Ph–tet]^−^ anion and a reduced stabilization of the single-phase mixture through ion solvation. As temperature increases, the system experiences a greater entropic driving force toward states of higher disorder and molecular randomness. This favors the release of water molecules from the hydration shell of the anion into the bulk liquid, where they possess greater translational freedom. In the case of [Ph–tet]^−^, the relatively small enthalpic contribution from anion–water interactions is insufficient to compensate for this entropy gain, leading to dehydration of the solvation shell. In contrast, the stronger hydration of [Bz]^−^ provides sufficient enthalpic stabilization to better retain water within its solvation environment. This promotes the displacement of water from solvent-separated ions, allowing the formation of more cation–anion aggregates characteristic of ionic liquids while the expelled water forms an excess “bulk-water” phase. The closest IL + water system previously studied is [P_4444_]­[CF_3_CO_2_] which also has LCST behavior.^47^ It was concluded that the IL exhibited strongly hydrophobic characteristic, and it was proposed that this resulted in poor dissociation of ion-pairs even in water-rich compositions, consistent with the comparative enthalpies of mixing of the [P_4444_]­[Bz] and [P_4444_]­[Ph–tet] systems with water here.

Desolvation and phase separation of IL/water mixtures can also be induced by salting-out more hydrophobic ion-pairs, typically the desired organic cation and less water structuring anion, from electrolyte solutions^2^ with the critical temperature for separation reducing as the concentration of the ions retained in the aqueous phase increases. Consequentially, changing the concentration of NaCl in ternary [P_4444_]­[Ph–tet]/water/NaCl mixtures (from “pure water” through seawater simulant to concentrated brine analogues) reduces the LCST point from 37, to 30, to 12 °C, as the increased ionic strength of the aqueous phase enhances salting-out of the more hydrophobic ion-pair. Mahfouz et al.^48^ recently described the LCST behavior of ternary IL/water mixtures containing two ILs and the impact on the critical temperature and osmotic strength of the solutions. Enhanced osmolality appears to be governed by the presence of “free” solvated ions in solution and can be partially decoupled from the induction of the LCST-phase transition that is driven by the more “hydrophobic” ion-pair desolvating from water.

Excess enthalpies for a range of IL/H_2_O binary mixtures have also been predicted using COSMO-RS.^49,50^ For observably *hydrophobic* ILs, such as 1-butyl-3-methylimidazolium trifluoromethylsulfonate, a small and positive excess enthalpy was predicted and miscibility was proposed to be an endothermic, entropically driven process. Here, ITC enables quantitative analysis of the hydration of ionic liquids by directly probing the enthalpy of solvation and so assessing the relative interactions of two structurally similar ionic liquids, [P_4444_]­[Ph–tet] and [P_4444_]­[Bz]. While the former exhibits LCST behavior, the latter remains miscible with water in larger temperature and composition ranges. The thermodynamics of water addition to these IL/water mixtures were quantified to elucidate how the balance between enthalpic and entropic contributions to the Gibbs free energy governs the phase boundaries. The pronounced nonideal behavior measured for the [P_4444_]­[Bz]/H_2_O system is characteristic of strong ion–water interactions. This is potentially associated with the formation of clathrate-like hydrates and is reflected in the larger negative enthalpy of mixing compared to that determined with the [Ph–tet]^−^ anion. The possible formation of salt clathrate hydrates^33–35^ between [P_4444_]­[Bz] and excess H_2_O needs a more detailed determination of the excess Gibbs free energy that was not possible in the present case. In the most basic terms, this indicates that mixing of [P_4444_]­[Ph–tet], which displays LCST characteristics, with water even at 25 °C is enthalpically less favorable than for the benzoate analogue.

## Conclusions

The importance of understanding the nature of water–IL interactions is especially important, not just for understanding the underpinning mechanisms behind temperature induced liquid/liquid equilibria^7–14^ but also in the context of new and emerging “water-in-ionic liquid” electrolytes for energy applications^51–54^ where the presence, and nature, of water within the ILs is critical to performance characteristics.

Two structurally similar ionic liquids, [P_4444_]­[Ph–tet] (LCST-forming) and [P_4444_]­[Bz] (fully water-miscible), were investigated calorimetrically. ITC, used here for the first time to study IL/H_2_O LCST systems, showed that the [P_4444_]­[Ph–tet]/H_2_O system exhibits a much smaller negative enthalpy of mixing (Δ_mix_ *H*), indicating intrinsically weak hydration that is easily disrupted upon heating. In the most basic terms, this indicates that mixing of [P_4444_]­[Ph–tet], which displays LCST characteristics, with water even at 25 °C is less favorable than for the benzoate analogue. Thus demixing of IL and water, enabling formation of a biphasic mixture, should be more favorable for the [Ph–tet]^−^ anion than for [Bz]^−^ and allows the general, and qualitative, description of “hydrophobicity” of the anion in the IL ion-pair to be quantified in terms of the relative enthalpy of mixing with water. These results are consistent with previous studies by Nitta et al.^47^ on [P_4444_]­[CF_3_CO_2_]/H_2_O mixtures where a small excess enthalpy of mixing was found for this LCST system and provides the missing experimental thermodynamic validation for earlier theoretical work.^9,17,18,55–58^

Neutron scattering demonstrated that [P_4444_]­[Ph–tet] forms poorly defined anion hydration shells, with water molecules dispersed within the ionic matrix. This structurally diffuse hydration correlates with the small negative Δ_mix_ *H* relative to [P_4444_]­[Bz], where water is more strongly bound. As temperature increases, these weak hydration shells break, increasing entropy and favoring hydrophobic cation–anion association in the IL-rich phase while releasing water into the water-rich phase—consistent with entropically driven LCST separation.^16–18,22,57,58^

This increase in “hydrophobicity” of the ion-pair, quantifiable—or at least rankable—through the ITC measurements can be used to understand whether an IL ion-pair is likely to preferentially self-associate in water, leading to formation of a hydrophobic IL and potentially to LCST behavior, or whether the ions gain sufficient excess enthalpy through solvation to retain a single continuous IL-water phase. In turn, this offers a methodology to study, and in turn, rationally tune the critical phase transition temperatures of IL/water mixtures as draw fluids to match the requirements of low-grade, sustainable heat sources such as solar or waste heat.

## Supplementary Material



## Notes

Experimental details of synthesis and characterization of 5-[^2^H_5_]­phenyl-1*H*-tetrazole, tetrabutylphosphonium 5-phenyl-tetrazolate and benzoate salts, and attempted deuteration of tetrabutylphosphonium bromide to [P_4444_–d_36_]­Br; isothermal titration calorimetric data; derivation and extraction of excess partial molar enthalpy data from the experimental colorimetry measurements; partial molars excess enthalpy of components as a function of composition; fitting to Legendre polynomials to extract behaviors and order of magnitude of the mixing enthalpy; experimental enthalpy of fusion data for [P_4444_]­[Bz] and experimental isothermal dissolution and titration calorimetric data for [P_4444_]­[Ph–tet]/H_2_O and [P_4444_]­[Bz]/H_2_O mixtures; and additional partial radial distribution functions from the EPSR model of the [P_4444_]­[Ph–tet]/H_2_O neutron scattering data (PDF)
