# Supplementary material for: Weak Anion Hydration Drives LCST Behavior in Ionic Liquid–Water Mixtures: Insights from Calorimetry and Neutron Diffraction
Source: J Phys Chem B. 2026 Oct 11;130(42):11256–67. doi: 10.1021/acs.jpcb.6c04727 (PMC13700004; doi:10.1021/acs.jpcb.6c04727)
Supplement: Supplementary file 1 [file jp6c04727_si_001.pdf]

Supporting Information: Weak Anion Hydration Drives LCST Behaviour in Ionic Liquid–Water Mixtures: Insights from Calorimetry and Neutron Diffraction

Sanskrita Madhukailya,† Guillaume Depraetère,‡ Nithavong Cam,‡ Tristan G. A. Youngs,¶ James P. Tellam,¶ Margarida Costa Gomes,§ Leila Moura,† and John D. Holbrey*,†

†*The QUILL Research Centre, School of Chemistry and Chemical Engineering, Queen's University Belfast, David Keir Building, Stranmillis Road, Belfast BT9 5AG, U.K.*

‡*Laboratoire de Chimie, Laboratoire de Chimie, ENS Lyon, CNRS and Universiteé de Lyon, 46 allée d'Italie, 69364 Lyon, France*

¶*ISIS Neutron and Muon Source, Rutherford Appleton Laboratory, Harwell Science & Innovation Campus, Didcot, Oxfordshire OX11 0QX, U.K.*

§*Laboratoire de Chimie, Laboratoire de Chimie, ENS Lyon, CNRS and Universiteé de Lyon, 46 allé e d'Italie, 69364 Lyon, France*

E-mail: j.holbrey@qub.ac.uk

Supporting Information Available

Synthesis and Characterisation

Instrumentation

Nuclear Magnetic Resonance (NMR) spectroscopic data were recorded using a Bruker Avance 400 MHz spectrometer. All chemical shifts (δ) are reported in parts per million (ppm), calibrated downfield from TMS using the residual solvent signal from $(CD_3)_2SO$ (δ 2.49 ppm septet for ^{1}H and δ 39.7 ppm for ^{13}C-NMR spectroscopy).

Deuteriated 5-[$^2H_5$]phenyl-1*H*-tetrazole

Deuteriated 5-[$^2H_5$]phenyl-1*H*-tetrazole was synthesised from bromobenzene-d_5 in two steps using the Rosenmund-von Braun reaction to form benzonitrile-d_5 followed by [2+3] cyclisation of the resultant benzonitrile-d_5 with NaN_3 and $[NH_4]Cl$ in N,N-dimethylformamide as described in the literature[1] by Zhao.

Benzonitrile-d_5 A three-neck round-bottom flask equipped with a reflux condenser and nitrogen inlet was charged with bromobenzene-d_5 (20.0 g, 1.00 equiv), copper(I) cyanide (12.9 g, 1.17 equiv), and N,N-dimethylformamide (20 mL). The reaction mixture was heated to reflux under nitrogen for 22 h. After cooling to room temperature, the mixture was quenched with aqueous ammonia and extracted with diethyl ether. The combined organic layers were washed successively with 6 M HCl and water, dried over anhydrous $MgSO_4$, filtered, and concentrated under reduced pressure. Purification by silica gel column chromatography (20:1 hexanes/EtOAc) afforded the product, benzonitrile-d_5, as a colorless oil (7.55 g, 57%).

5-[$^2H_5$]Phenyl-1*H*-tetrazole A mixture of benzonitrile-d_5 (7.55 g, 1.00 equiv), sodium azide (5.23 g, 1.15 equiv), and ammonium chloride (4.11 g, 1.10 equiv) in DMF (50 mL) was heated at 120 °C for 20 h. The reaction mixture was cooled to room temperature, and the solvent was removed under reduced pressure. The residue was poured into D_2O (20 mL) to afford the crude

tetrazole. The material was dissolved in additional D_2O and heated at 80 °C for 6 h, then brought to reflux and subsequently cooled to allow crystallization. The resulting solid, 5-[$^2H_5$]Phenyl-1*H*-tetrazole was collected as a white crystalline powder.

Tetrabutylphosphonium 5-phenyl-tetrazolate and benzoate

Tetrabutylphosphonium 5-phenyltetrazolate salts (with either hydrogenous or perdeuterated anions, $[C_7N_4H_5]^-$ and $[C_7N_4D_5]^-$ respectively) synthesised following the acid-base neutralisation method, as reported by Moura et al.[2] from the neutral 5-phenyltetrazole and tetrabutylphosphonium hydroxide solution (40 wt% in H_2O, 18.9 g, 1 eq) with 5-phenyl-tetrazole (12.68 g, 0.087 mol). After addition of the two reagents, the mixture was stirred at 60 °C for 4 hr, forming a colourless biphasic mixture. The two layers are transferred into a separating flask after which the IL-rich layer was extracted with ethyl acetate, washed with water three times to remove unreacted reactants (tetrabutylphosphonium hydroxides) and the organic solvent was then evaporated under reduced pressure, forming a colourless viscous liquid, which crystallised to a colourless solid upon standing and was dried further under reduced pressure.

$[P_{4444}]$[Ph−tet]: MS, ^1H, ^{13}C, and ^{31}P NMR spectra, and melting point (74-75 °C) were consistent with those previously reported. ^1H (400 MHz, DMSO-d_6) δ (ppm): 7.97 (2H, d, phenyl), 7.36 (2H, m, phenyl), 7.23 (1H, t, phenyl), 2.16 (8H, m, P-C**H**$_2$-), 1.38 (16H, m, P-CH$_2$-(C**H**$_2$)$_2$-), 0.87 (12H, m, P-(CH$_2$)$_3$-C**H**$_3$); ^{13}C(100.55 MHz; DMSO-d_6): 160.53 (N-C-N), 132.73-125.56 (4 peaks, phenyl ring), 23.31 (d, P-**C**H$_2$-), 22.62 (doublet, P-CH$_2$-**C**H$_2$-), 17.50 (d, P-(CH$_2$)$_2$-**C**H$_2$-), 13.06 (P-(CH$_2$)$_3$-**C**H$_3$). $^{31}P\{1H\}$ (161.92 MHz, DMSO-d_6) δ (ppm) = 33.78 (s, **P**, $[P_{4444}]^+$).

$[P_{4444}]$[Bz] was prepared in an identical manner from tetrabutylphosphonium hydroxide solution (40 wt% in H_2O) and benzoic acid, and was dried under vacuum to yield a colourless hygroscopic solid that was stored under an inert, dry atmosphere. $[P_{4444}]$[Bz]: ^1H, ^{13}C, and $^{31}P\{1H\}$ NMR spectra, and melting point (39.49 °C, Table S3) were consistent with those previously reported from Ando et al.[3] and Gulotty et al.[4]

^{1}H (400 MHz, $CDCl_3$) δ (ppm): 8.07-8.04 (2H, m, phenyl), 7.30-7.26 (3H, m, phenyl), 2.23 (8H, m, P-C**H**$_2$-), 1.45 (16H, m, P-CH_2-(C**H**$_2$)$_2$-), 0.91 (12H, m, P-$(CH_2)_3$-C**H**$_3$); ^{13}C NMR (100.55 MHz; $CDCl_3$) δ 168.86, 135.94, 131.94, 130.43, 129.09, 127.48, 24.0, 23.9, 18.9, 13.5; ^{31}P{1H} (161.92 MHz; $CDCl_3$) δ 33.64;

Attempted deuteration of tetrabutylphosphonium bromide to [$P_{4444}-d_{36}$]Br

Deuteriation of [P_{4444}]Br was attempted using a procedure reported by Sajiki and co-workers,[5] wherein a series of long-chain fatty acids were deuterated by employing a mixed solvent system of D_2O/*i*PrOH (deuterium oxide and iso-propanol) with a 10% platinum on carbon (Pt/C) catalyst in a sealed pressure vessel and utilises *in-situ* generation of H_2 from isopropanol to activate the catalyst, forming acetone as a biproduct.[6] This method was also identical to that reported for the deuteration of alkyltrimethylammonium chlorides.[7,8]

Initially synthesis screening was carried out on a small scale in an AntonPaar Monowave microwave reactor at a substrate concentration of 0.25 mmol (0.0848 g), 8 mol% (0.004 g) of the catalyst (10% Pt/C) with a D_2O/*i*PrOH ratio of 3/0.5 ml at 120 °C for 24 hr, replicating Sajiki's procedure.[5] At the end of 24 hr the resulting mixture was first subjected to solvent evaporation and the remaining crude product was then analysed using mass spectrometer. The results showed only a 2.7% conversion (m/z 260.61, 100%) into its deuterated form (one out of 36 hydrogens substituted by deuterium. The reaction was repeated following the same method for 72 hr, but no significant conversion of H to D was observed. Then, the synthesis was re-attempted with a slight modification. The product obtained after 24 hours was reused in a consecutive reaction batch in the microwave, with a fresh mixture of solvents (D_2O/*i*PrOH) and catalyst system, maintaining the same quantities as used initially. At the end of another 24 hr (48 hr in total), the reaction resulted in 5.5% conversion (m/z 261.24 (100%)) of [P_{4444}]Br into its partially deuterated form. This was repeated three times for a total of 120 hr yielding a substitution of 17 hydrogens with deuteriums from the original substrate salt.

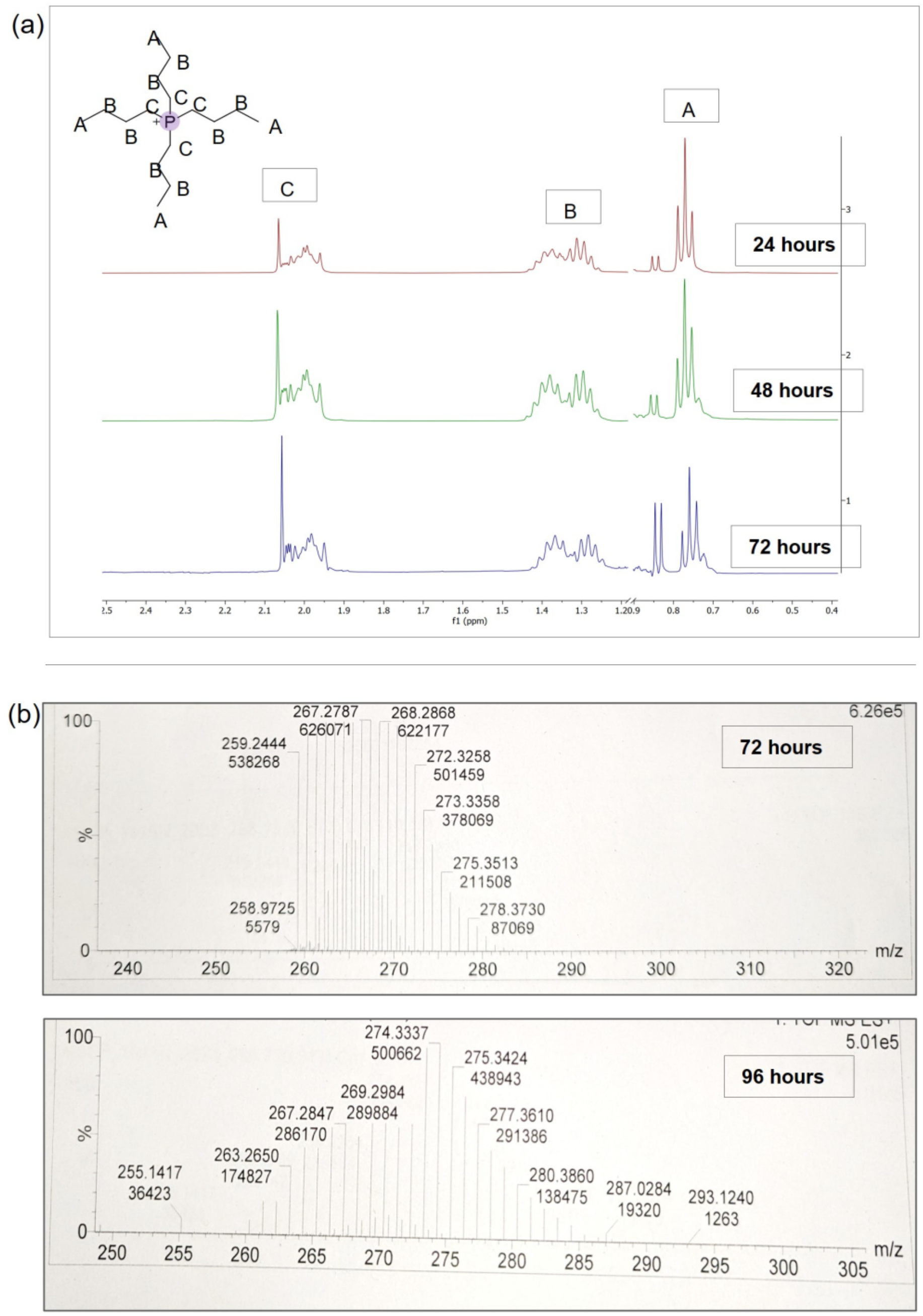

Figure S1: Progress of the deuteration reaction for $[P_{4444}]Br$ from (a) 1H-NMR spectra is shown here, with a singlet acetone peak at 2.07 ppm. Sample aliquots were collected after 24, 48 and 72 hours, (b) Mass spectra showing H-D conversion reaction for $[P_{4444}]Br$ after 72 hr (top) of the deuteration reaction (in *i*PrOH/D_2O, with 8 mol% Pt/C) with m/z value of 267.27 at 100%, and 96 hr (bottom) with m/z value 100% at 274.34. Both these samples for MS were collected post work-up of the reaction mixtures.

The progress of the reaction was monitored *via* mass spectroscopic studies and the data

are shown in Fig. S1. A summary of the observations are shown in Table S1. Interestingly, methods like continuous-flow hydrogen-deuterium exchange reactions involving the concept of iterative runs of catalytic deuteration, using flow reactors have also been reported.[9,10] Sajiki and co-workers,[7] who attempted the deuteration of alkylammonium salts, observed that the α-hydrogens (adjacent to the ammonium cationic centre) were not deuterated using the method they employed. This regioselectivity issue was also reported in the deuteration of amines using metal nanoparticles (NPs), which were more prone to label the β-positions more effectively.[11] In contrast, McGrogan et al.[12] confirmed that quicker and initial deuterations selectively occurred on the α-hydrogens, as evidenced by mass spectroscopy and ^{1}H-NMR spectroscopy results.

Table S1: Amount of hydrogen to deuterium conversion recorded during the deuteration of [P_{4444}]Br, involving addition of fresh batches of catalyst (8 mol% Pt/C) and solvent-systems (D_2O/*i*PrOH at 3/0.5 ml) after every 24 hr, with the same substrate collected post work-up of the reaction mixture.

Time (h)	Progress (m/z)	Conversion (%)	D-content
24	260.61 (100%)	2.7	$C_{16}H_{35}D_1P$
48	261.24 (100%)	5.5	$C_{16}H_{34}D_2P$
72	267.27 (100%)	22.2	$C_{16}H_{28}D_8P$
96	274.34 (100%)	38.8	$C_{16}H_{22}D_{14}P$
120	278.37 (100%)	47.2	$C_{16}H_{19}D_{17}P$

Further studies supporting these observations on other compounds, have also been reported in the literature.[9] In this study, however, no noticeable deuterium labeling at the α-positions was observed, as the ^{1}H-NMR spectra did not show a decrease in the α-H peaks, even after 72 hours of the reaction (Fig. S1(a)). Nevertheless, the corresponding mass spectra for comparison display significant levels of deuteration, as discussed above.

For upscaling the reaction, a larger substrate concentration (2.5 mmol, 0.8483 g) was used, along with an equivalent solvent ratio of D_2O/*i*PrOH (20/4 ml). The same procedure of reusing the product batch was followed. However, the reaction did not proceed to completion, with no single hydrogen getting converted to deuterium. Multiple attempts were made by reducing the substrate concentration while keeping the solvent ratio constant, and by increasing the catalyst loading, yet no significant conversion of H to D was observed.

Table S2: Amount of hydrogen to deuterium conversion recorded during the deuteration of $[P_{4444}]Br$, uisng the same batches of catalyst (8 mol% Pt/C), solvent-systems (D_2O/*i*PrOH at 3/0.5 ml) and substrate ($[P_{4444}]Br$), omitting the extraction and purification steps after every 24 hr.

Time (h)	Progress (m/z)	D-content
24	260.24 (100%)	$C_{16}H_{35}D_1P$
48	259.23 (100%)	$C_{16}H_{36}D_0P$
72	260.23 (100%)	$C_{16}H_{35}D_1P$
96	261.23 (100%)	$C_{16}H_{34}D_2P$
120	262.23 (100%)	$C_{16}H_{33}D_3P$
144	263.24 (100%)	$C_{16}H_{32}D_4P$
168	263.24 (100%)	$C_{16}H_{32}D_4P$

In a simultaneous set of reactions, extraction and purification between every 24 hr was omitted and a small aliquot of the sample was analysed by MS each time to check the progress. The results showed an initial decrease in D-content, followed by a small increase, and finally, a plateau by the end of 168 hr, using the same batch of reaction materials (Table S2). Despite the stable isotope effect of deuterium,[13] the lack of progress in the reaction could be attributed to a D-H exchange reaction (reverse reaction), which may occur due to the presence of *i*PrOH as a hydrogen source, as discussed by Yamada et al.[14]

The low yield of partially deuterated $[P_{4444}]Br$ at the conclusion of each reaction cycle was insufficient to provide the required quantity for neutron scattering experiments. As a result, the neutron scattering experiments were ultimately conducted using hydrogenous $[P_{4444}]^+$ cations, and introducing H/D isotopic contrast through anions and water.

Isothermal titration calorimetric data

Excess partial molar enthalpy

Considering the injection of a pure component, the relationships between $\delta Q1$, and the partial molar excess enthalpies of the components in the mixtture, described by the relationship (eq. 4 in paper):

$$\begin{aligned}\delta Q = \delta n_1 &\left[\bar{H}_1^E\left(x_{\text{cell}}^{\text{fin}}, T\right) - \bar{H}_1^E\left(x_{\text{syr}}, T\right)\right]\\ +\delta n_2 &\left[\bar{H}_2^E\left(x_{\text{cell}}^{\text{fin}}, T\right) - \bar{H}_2^E\left(x_{\text{syr}}, T\right)\right]\\ +n_1 &\left[\bar{H}_1^E\left(x_{\text{cell}}^{\text{fin}}, T\right) - \bar{H}_1^E\left(x_{\text{cell}}^{\text{ini}}, T\right)\right]\\ +n_2 &\left[\bar{H}_2^E\left(x_{\text{cell}}^{\text{fin}}, T\right) - \bar{H}_2^E\left(x_{\text{cell}}^{\text{ini}}, T\right)\right]\end{aligned}$$

simplifies to equation S1 as follows,

$$\begin{aligned}\delta Q = \delta n_1 &\left[\bar{H}_1^\ell\left(x_{\text{cell}}^{\text{fin}}, T\right) - \bar{H}_1^{\ell,*}(T)\right]\\ +n_1 &\left[\bar{H}_1^\ell\left(x_{\text{cell}}^{\text{fin}}, T\right) - \bar{H}_1^\ell\left(x_{\text{cell}}^{\text{ini}}, T\right)\right]\\ +n_2 &\left[\bar{H}_2^\ell\left(x_{\text{cell}}^{\text{fin}}, T\right) - \bar{H}_2^\ell\left(x_{\text{cell}}^{\text{ini}}, T\right)\right]\end{aligned} \tag{S1}$$

Since mixing enthalpy is defined by equation S2, therefore, substituting it in equation S1, δQ can be re-written as S3,

$$\begin{aligned}n\Delta_{\text{mix}}H(x,T) =& n_1\left[\bar{H}_1^\ell(x,T) - H_1^{\ell,*}(T)\right]\\ &+n_2\left[\bar{H}_2^\ell(x,T) - H_2^{\ell,*}(T)\right]\end{aligned} \tag{S2}$$

$$\begin{aligned}\delta Q =& \delta n_1\left[\bar{H}_1^\ell\left(x_{\text{cell}}^{\text{fin}}, T\right) - \bar{H}_1^{\ell,*}(T)\right]\\ &+ n_1\left[\bar{H}_1^\ell\left(x_{\text{cell}}^{\text{fin}}, T\right) - \bar{H}_1^{\ell,*}(T)\right] - n_1\left[\bar{H}_1^\ell\left(x_{\text{cell}}^{\text{ini}}, T\right) - \bar{H}_1^{\ell,*}(T)\right]\\ &+ n_2\left[\bar{H}_2^\ell\left(x_{\text{cell}}^{\text{fin}}, T\right) - \bar{H}_2^{\ell,*}(T)\right] - n_2\left[\bar{H}_2^\ell\left(x_{\text{cell}}^{\text{ini}}, T\right) - \bar{H}_2^{\ell,*}(T)\right]\\ =& (n_1 + \delta n_1 + n_2)\Delta_{\text{mix}}H\left(x_{\text{cell}}^{\text{fin}}, T\right) - (n_1 + n_2)\Delta_{\text{mix}}H\left(x_{\text{cell}}^{\text{ini}}, T\right)\end{aligned} \tag{S3}$$

Dividing equation S3 by $\delta n_1 \ll n_1 + n_2$ produces the partial derivative of the mixing enthalpy

with respect to n_1 at constant n_2, T, p, as shown in equation S4. This is, by definition, the excess partial molar enthalpy of component 1.

$$\frac{\delta Q_{\mathrm{ITC}}}{\delta n_1} = \left(\frac{\partial\left(n\Delta_{\mathrm{mix}}H(x_{\mathrm{cell}},T)\right)}{\partial n_1}\right)_{n_2,p,T} = H_1^E\left(x_{\mathrm{cell}},T\right) \tag{S4}$$

In this same way, for component 2 involving dissolution experiment, equation S1 becomes equation S5 as,

$$\begin{aligned} \delta Q = \delta n_2 &\left[\bar{H}_2^{\ell}\left(x_{\mathrm{cell}}^{\mathrm{fin}},T\right) - \bar{H}_2^{s,*}\left(T\right)\right] \\ &+n_1\left[\bar{H}_1^{\ell}\left(x_{\mathrm{cell}}^{\mathrm{fin}},T\right) - \bar{H}_1^{\ell}\left(x_{\mathrm{cell}}^{\mathrm{ini}},T\right)\right] \\ &+n_2\left[\bar{H}_2^{\ell}\left(x_{\mathrm{cell}}^{\mathrm{fin}},T\right) - \bar{H}_2^{\ell}\left(x_{\mathrm{cell}}^{\mathrm{ini}},T\right)\right] \end{aligned} \tag{S5}$$

Thus, from fusion enthalpy $\Delta_{\mathrm{fus}}H_2\left(T\right) = \bar{H}_2^{\ell,*}\left(T\right) - \bar{H}_2^{s,*}\left(T\right)$, the excess partial molar enthalpy of component 2, is given by,

$$\frac{\delta Q_{\mathrm{dissolution}}}{\delta n_2} = H_2^E\left(x_{\mathrm{cell}},T\right) + \Delta_{\mathrm{fus}}H_2\left(T\right) \tag{S6}$$

Mixing enthalpy

Figure S2 shows the partial molars excess enthalpy of both the component of the two IL-water mixtures as a function of composition in IL. Direct experimental estimation of partial molar excess enthalpy (orange and blue points) act as guards to ensure the fit is consistent with actual values. Here, first order Legendre polynomials (2 fitting parameters) is used to ensure that no overfitting of the experimental data is carried out. Fittings to second and third order Legendre polynomials (3 and 4 fitting parameters) gave similar behaviours of the partial molar excess enthalpies, and most importantly the similar order of magnitude for mixing enthalpy, as shown on Figure S3.

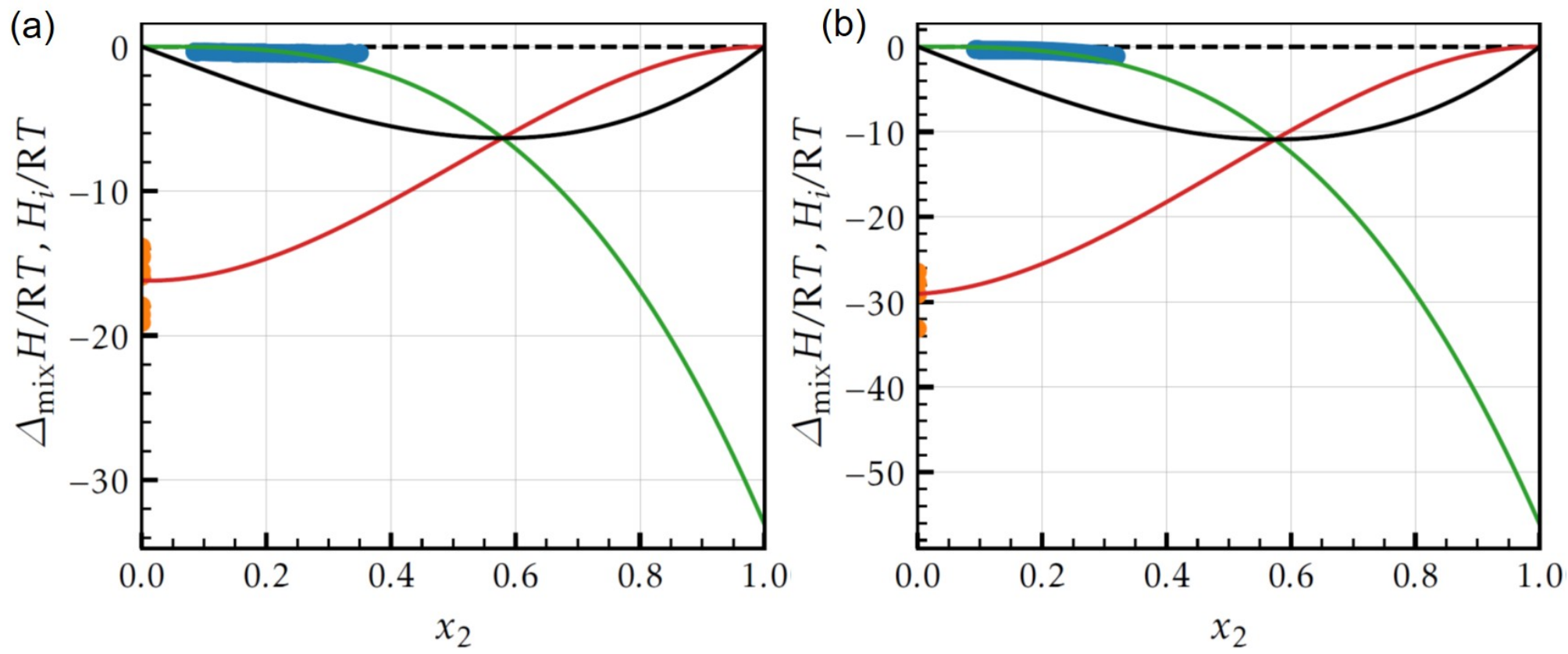

Figure S2: Partial molar excess enthalpies and mixing enthalpy obtained from ITC experiment and fitted to 2 parameters Legendre polynomials for (a) (1) H_2O + [P_{4444}][Ph−tet] (2) and (b) (1) H_2O + [P_{4444}][Bz] (2). green line H_1^E/RT, red line H_2^E/RT, black line H^E/RT, orange dot and blue dot experimental partial molar excess enthalpy

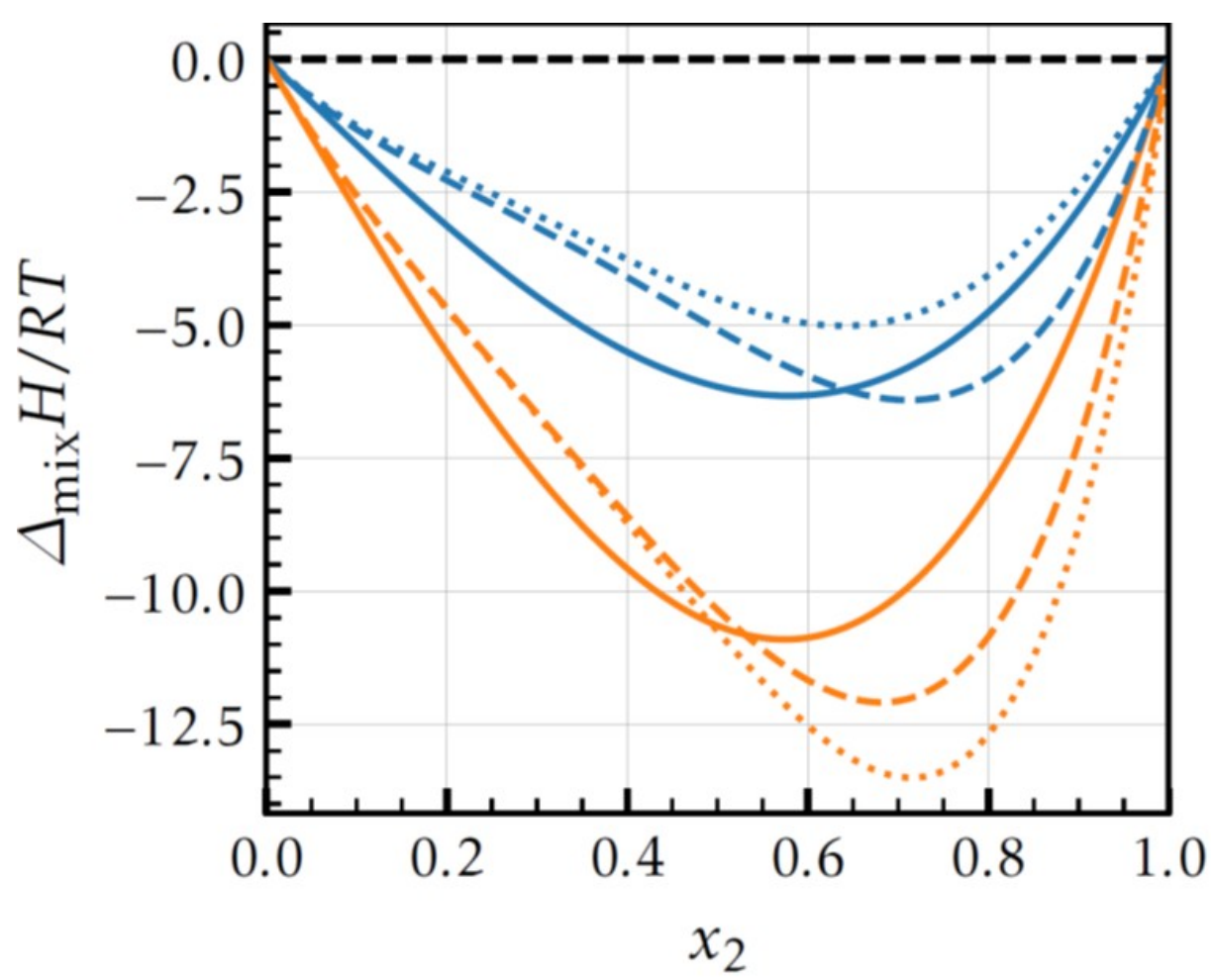

Figure S3: Mixing enthalpies of the (1) H_2O + [P_{4444}][Ph−tet] (2) (blue) and (1) H_2O + [P_{4444}][Bz] (2) (orange) calculated with different degrees of fitting with Legendre polynomials. The order of magnitude and relative positions of mixing enthalpy do not depend significantly on degree of the fitting. solid / solid order 1, dashed / dashed order 2, dotted / dotted order 3

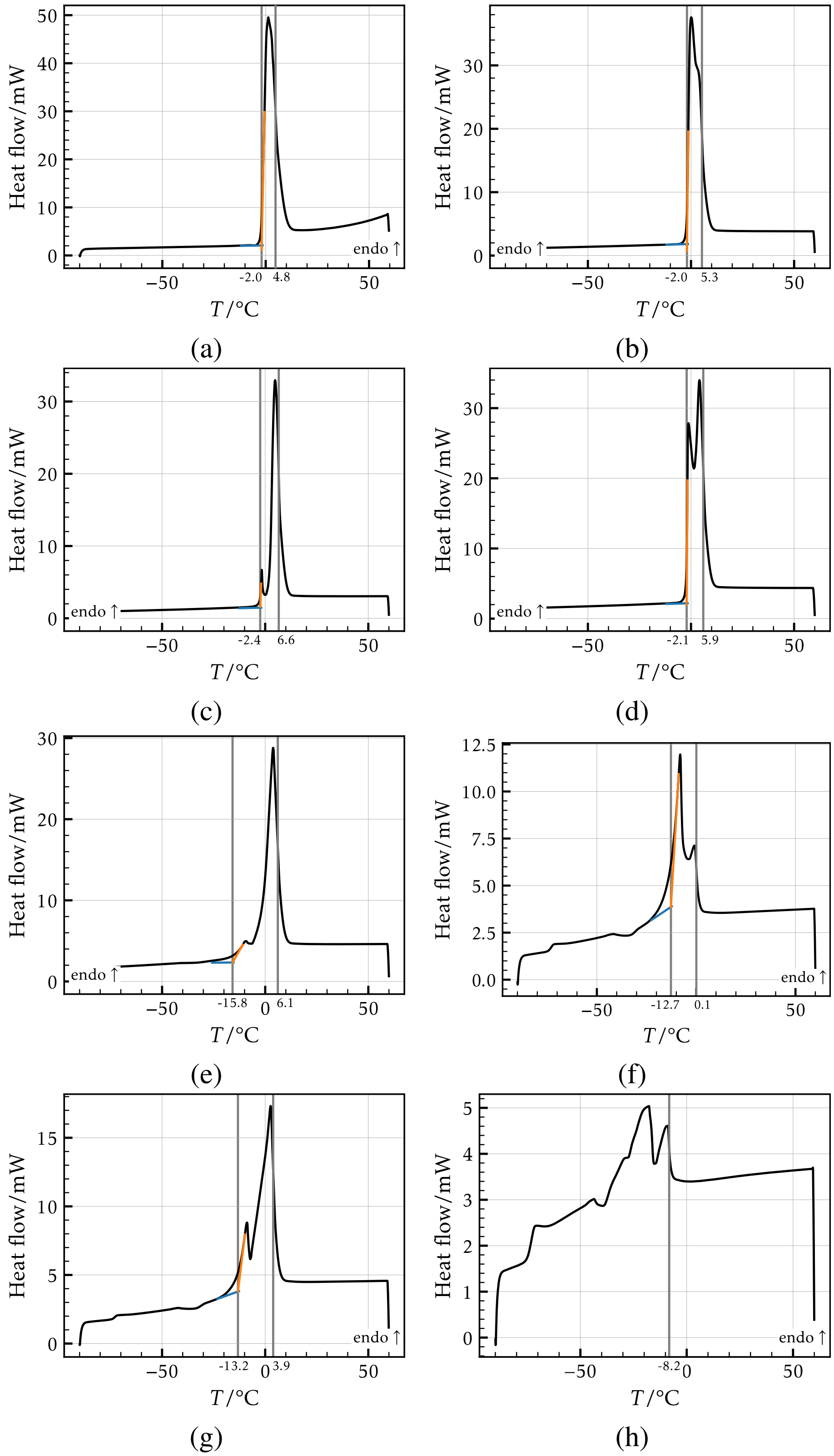

Figure S4: DSC heating step of the mixture $[P_{4444}][Bz]+H_2O$ at different compositions (scan rate 5 K min^{-1}) for (a) χ_{H_2O} = 0.005, (b) χ_{H_2O} = 0.013, (c) χ_{H_2O} = 0.020, (d) χ_{H_2O} = 0.030, (e) χ_{H_2O} = 0.045, (f) χ_{H_2O} = 0.066, (g) χ_{H_2O} = 0.099, (h) χ_{H_2O} = 0.159.

Enthalpy of fusion of the tetrabutylphosphonium benzoate

Table S3: DSC data for pure IL, tetrabutylphosphonium benzoate ($[P_{4444}][Bz]$). Q corresponds to integrated heat effect of the observed transition. For further treatment, the average value of 15.8 ± 3.0 kJ mol^{-1} for fusion enthalpy of $[P_{4444}][Bz]$ is used.

Mass /mg	Scan rate /K min^{-1}	Onset /°C	Q /mJ	Q /kJ mol^{-1}
1.965	20	36.1	101.34	19.62
3.865	20	39.0	165.39	16.28
1.965	10	36.0	76.23	14.76
2.070	10	37.7	80.63	14.82
3.865	10	39.3	161.56	15.91
3.865	10	42.8	174.45	17.17
3.865	10	42.3	210.70	20.74
1.965	5	22.6	54.80	10.61
3.865	5	40.5	134.05	13.20
3.865	5	38.6	151.69	14.93

Isothermal Dissolution and Titration Calorimetric data

Table S4: Isothermal titration calorimetry data for system (1) H_2O + $[P_{4444}][Ph{-}tet]$ (2) at 298.15 K. n_i is the mole quantity of i in the cell (subscript c) or in the syringe (subscript s) and Q is the integrated heat effect of the injection.

$n_{1,c}$ /mmol	$n_{2,c}$ /mmol	$n_{1,s}$ /μmol	$n_{2,s}$ /μmol	Q /mJ
3.475	1.739	54.178	0	-55.97
3.529	1.739	109.800	0	-128.38
3.639	1.739	109.800	0	-130.94
3.748	1.739	109.800	0	-133.78
3.858	1.739	109.800	0	-134.15
3.968	1.739	109.800	0	-133.32
4.078	1.739	109.800	0	-133.82
4.188	1.739	109.800	0	-135.34
4.297	1.739	109.800	0	-133.32

Continued on next page

Table S4: – continued from previous page

$n_{1,c}$ /mmol	$n_{2,c}$ /mmol	$n_{1,s}$ /μmol	$n_{2,s}$ /μmol	Q /mJ
4.407	1.739	109.800	0	-132.34
4.517	1.739	109.800	0	-133.37
4.627	1.739	109.800	0	-131.33
4.737	1.739	54.178	0	-60.51
4.791	1.739	54.178	0	-61.31
4.845	1.739	54.178	0	-59.90
4.899	1.739	54.178	0	-58.07
4.953	1.739	54.178	0	-58.46
5.008	1.739	54.178	0	-58.66
5.062	1.739	54.178	0	-59.02
5.116	1.739	54.178	0	-59.50
5.170	1.739	54.178	0	-59.18
5.224	1.739	54.178	0	-57.86
5.278	1.739	165.475	0	-194.84
5.444	1.739	165.475	0	-192.38
5.609	1.739	165.475	0	-194.53
5.775	1.739	165.475	0	-192.94
5.940	1.739	165.475	0	-190.96
6.106	1.739	165.475	0	-189.42
6.271	1.739	165.475	0	-187.26
6.437	1.739	165.475	0	-187.84
6.602	1.739	165.475	0	-187.18
6.768	1.739	165.475	0	-187.62
8.272	1.605	109.800	0	-123.49

Continued on next page

Table S4: – continued from previous page

$n_{1,c}$ /mmol	$n_{2,c}$ /mmol	$n_{1,s}$ /μmol	$n_{2,s}$ /μmol	Q /mJ
8.382	1.605	109.800	0	-123.87
8.492	1.605	109.800	0	-122.01
8.602	1.605	109.800	0	-124.27
8.712	1.605	109.800	0	-125.76
8.821	1.605	109.800	0	-123.39
8.931	1.605	109.800	0	-125.39
9.041	1.605	109.800	0	-124.48
9.151	1.605	109.800	0	-125.38
15.565	1.542	54.178	0	-51.79
15.619	1.542	54.178	0	-50.97
15.674	1.542	54.178	0	-51.07
15.728	1.542	54.178	0	-49.48
15.782	1.542	54.178	0	-49.99
15.836	1.542	54.178	0	-49.34
15.890	1.542	54.178	0	-49.75
15.944	1.542	54.178	0	-49.56
15.999	1.542	54.178	0	-49.58
16.053	1.542	54.178	0	-49.68
16.107	1.542	54.178	0	-49.56
16.161	1.542	54.178	0	-49.40
16.215	1.542	54.178	0	-49.83
16.270	1.542	54.178	0	-49.62
16.324	1.542	54.178	0	-49.40
16.378	1.542	54.178	0	-47.87

Continued on next page

Table S4: – continued from previous page

$n_{1,c}$ /mmol	$n_{2,c}$ /mmol	$n_{1,s}$ /μmol	$n_{2,s}$ /μmol	Q /mJ
16.432	1.542	54.178	0	-47.17
16.486	1.542	54.178	0	-48.27
16.540	1.542	54.178	0	-48.33
16.595	1.542	54.178	0	-48.01
2.982	1.605	109.800	0	-123.24
3.092	1.605	109.800	0	-142.27
3.202	1.605	109.800	0	-141.41
3.311	1.605	109.800	0	-136.45
3.421	1.605	109.800	0	-139.11
3.531	1.605	109.800	0	-135.28
3.641	1.605	109.800	0	-134.46
3.751	1.605	109.800	0	-136.23
3.860	1.605	109.800	0	-129.61
3.970	1.605	109.800	0	-132.02
4.080	1.605	109.800	0	-131.14
4.190	1.605	109.800	0	-126.93
4.300	1.605	109.800	0	-130.82
4.409	1.605	109.800	0	-127.86
4.519	1.605	109.800	0	-126.28
4.629	1.605	109.800	0	-129.65
4.739	1.605	109.800	0	-124.99
4.849	1.605	109.800	0	-126.86
4.958	1.605	109.800	0	-127.81
5.068	1.605	109.800	0	-122.65

Continued on next page

Table S4: – continued from previous page

$n_{1,c}$ /mmol	$n_{2,c}$ /mmol	$n_{1,s}$ /μmol	$n_{2,s}$ /μmol	Q /mJ
5.178	1.605	109.800	0	-128.41
5.288	1.605	109.800	0	-126.24
5.398	1.605	109.800	0	-125.43
5.507	1.605	109.800	0	-127.46
5.617	1.605	109.800	0	-122.80
5.727	1.605	109.800	0	-126.66
5.837	1.605	109.800	0	-125.31
5.947	1.605	109.800	0	-122.65
6.056	1.605	109.800	0	-125.83
6.166	1.605	109.800	0	-124.42
6.276	1.605	109.800	0	-123.65
6.386	1.605	109.800	0	-124.97
6.496	1.605	109.800	0	-122.02
6.605	1.605	109.800	0	-123.92
6.715	1.605	109.800	0	-124.78
6.825	1.605	109.800	0	-123.35
6.935	1.605	109.800	0	-126.58
7.045	1.605	109.800	0	-125.78
7.154	1.605	109.800	0	-123.59
7.264	1.605	109.800	0	-125.17
7.374	1.605	109.800	0	-124.23
7.484	1.605	109.800	0	-123.07
7.594	1.605	109.800	0	-124.45
7.703	1.605	109.800	0	-121.93

Continued on next page

Table S4: – continued from previous page

$n_{1,c}$ /mmol	$n_{2,c}$ /mmol	$n_{1,s}$ /μmol	$n_{2,s}$ /μmol	Q /mJ
7.813	1.605	109.800	0	-125.58
7.923	1.605	109.800	0	-125.33
8.033	1.605	109.800	0	-119.85
5.563	1.365	109.800	0	-110.63
5.672	1.365	109.800	0	-110.98
5.782	1.365	109.800	0	-111.31
5.892	1.365	109.800	0	-109.87
6.002	1.365	109.800	0	-110.64
6.112	1.365	275.275	0	-281.13
6.387	1.365	275.275	0	-275.53
6.662	1.365	275.275	0	-275.74
6.937	1.365	275.275	0	-275.71
7.213	1.365	275.275	0	-275.45
7.488	1.365	275.275	0	-275.98
7.763	1.365	275.275	0	-275.98
8.039	1.365	275.275	0	-273.68
8.314	1.365	275.275	0	-272.87
8.589	1.365	275.275	0	-270.67
8.864	1.365	109.800	0	-104.10
8.974	1.365	109.800	0	-105.05
9.084	1.365	109.800	0	-104.63
9.194	1.365	109.800	0	-104.35
9.304	1.365	109.800	0	-104.90
9.413	1.365	275.275	0	-266.83

Continued on next page

Table S4: – continued from previous page

$n_{1,c}$ /mmol	$n_{2,c}$ /mmol	$n_{1,s}$ /μmol	$n_{2,s}$ /μmol	Q /mJ
9.689	1.365	275.275	0	-267.08
9.964	1.365	275.275	0	-262.47
10.239	1.365	275.275	0	-261.07
10.514	1.365	275.275	0	-260.10
10.790	1.365	275.275	0	-257.43
11.065	1.365	275.275	0	-255.21
11.340	1.365	275.275	0	-252.22
11.616	1.365	275.275	0	-250.52
11.891	1.365	275.275	0	-248.12
12.166	1.365	109.800	0	-95.58
12.276	1.365	109.800	0	-96.13
12.386	1.365	109.800	0	-95.98
12.496	1.365	109.800	0	-96.09
12.605	1.365	109.800	0	-96.30

Table S5: Dissolution calorimetry data for system (1) H_2O + [P_{4444}][Ph−tet] (2) at 298.15 K. n_i is the mole quantity of i in the cell (subscript c) or in the cartridge (subscript d for dispenser) and Q_{sol} is the integrated heat effect of the dissolution (taking into account heat effect of the reference cartridge).

$n_{1,c}$ /mmol	$n_{2,c}$ /mmol	$n_{1,d}$ /μmol	$n_{2,d}$ /μmol	Q_{sol} /mJ
914.005	0	0	53.561	-1384.53
914.005	0.054	0	15.382	-320.75
916.070	0	0	24.693	-710.83
916.070	0.025	0	36.911	-639.46
914.388	0	0	18.489	-506.38
914.388	0.018	0	26.621	-419.57
919.374	0	0	3.881	-68.21
919.374	0.004	0	5.092	-101.05

Table S6: Isothermal titration calorimetry data for system (1) H_2O + $[P_{4444}]$[Bz] (2) at 298.15 K. n_i is the mole quantity of i in the cell (subscript c) or in the syringe (subscript s) and Q is the integrated heat effect of the injection.

$n_{1,c}$ /mmol	$n_{2,c}$ /mmol	$n_{1,s}$ /μmol	$n_{2,s}$ /μmol	Q /mJ
49.804	0.003	69.150	3.262	-108.65
49.874	0.007	69.150	3.262	-109.31
49.943	0.010	69.150	3.262	-108.82
50.012	0.013	69.150	3.262	-107.70
50.081	0.016	69.150	3.262	-108.07
50.150	0.020	69.150	3.262	-106.68
50.219	0.023	69.150	3.262	-106.60
50.288	0.026	69.150	3.262	-104.04
50.358	0.029	69.150	3.262	-104.39
50.427	0.033	69.150	3.262	-103.00
50.496	0.036	69.150	3.262	-103.47
50.565	0.039	69.150	3.262	-104.53
50.634	0.042	69.150	3.262	-102.17
50.703	0.046	69.150	3.262	-100.38
50.772	0.049	69.150	3.262	-101.05
50.842	0.052	69.150	3.262	-99.74
50.911	0.055	69.150	3.262	-100.24
50.980	0.059	69.150	3.262	-98.95
51.049	0.062	69.150	3.262	-99.04
15.045	1.656	54.178	0	-47.21
15.099	1.656	54.178	0	-44.13
15.153	1.656	54.178	0	-47.20

Continued on next page

Table S6: – continued from previous page

$n_{1,c}$ /mmol	$n_{2,c}$ /mmol	$n_{1,s}$ /μmol	$n_{2,s}$ /μmol	Q /mJ
15.207	1.656	54.178	0	-47.21
15.261	1.656	54.178	0	-47.10
15.316	1.656	54.178	0	-46.78
15.370	1.656	54.178	0	-48.55
15.424	1.656	54.178	0	-47.35
15.478	1.656	54.178	0	-47.81
15.532	1.656	54.178	0	-47.25
15.586	1.656	54.178	0	-46.45
15.641	1.656	54.178	0	-45.78
15.695	1.656	54.178	0	-45.65
15.749	1.656	54.178	0	-46.57
15.803	1.656	54.178	0	-45.54
15.857	1.656	54.178	0	-47.89
15.912	1.656	54.178	0	-45.60
15.966	1.656	54.178	0	-44.14
16.020	1.656	54.178	0	-45.85
16.074	1.656	54.178	0	-45.44
16.128	1.656	54.178	0	-44.48
16.182	1.656	54.178	0	-44.91
16.237	1.656	54.178	0	-43.86
3.825	1.796	137.637	0	-380.83
3.963	1.796	137.637	0	-358.44
4.101	1.796	137.637	0	-348.05
4.238	1.796	137.637	0	-324.89

Continued on next page

Table S6: – continued from previous page

$n_{1,c}$ /mmol	$n_{2,c}$ /mmol	$n_{1,s}$ /μmol	$n_{2,s}$ /μmol	Q /mJ
4.376	1.796	137.637	0	-317.90
4.514	1.796	137.637	0	-299.27
4.651	1.796	137.637	0	-288.31
4.789	1.796	137.637	0	-273.65
4.927	1.796	137.637	0	-266.40
5.064	1.796	137.637	0	-252.67
5.202	1.796	137.637	0	-245.13
5.339	1.796	137.637	0	-236.68
5.477	1.796	137.637	0	-228.88
5.615	1.796	137.637	0	-222.60
5.752	1.796	137.637	0	-213.81
5.890	1.796	137.637	0	-206.76
6.028	1.796	137.637	0	-201.79
6.165	1.796	137.637	0	-196.08
6.303	1.796	137.637	0	-191.10
6.441	1.796	137.637	0	-185.23
6.578	1.796	137.637	0	-183.28
6.716	1.796	137.637	0	-179.69
6.853	1.796	137.637	0	-175.81
6.991	1.796	137.637	0	-172.33
7.129	1.796	137.637	0	-169.62
7.266	1.796	137.637	0	-166.87
7.404	1.796	137.637	0	-164.77
7.542	1.796	137.637	0	-162.77

Continued on next page

Table S6: – continued from previous page

$n_{1,c}$ /mmol	$n_{2,c}$ /mmol	$n_{1,s}$ /μmol	$n_{2,s}$ /μmol	Q /mJ
7.679	1.796	137.637	0	-162.86
7.817	1.796	137.637	0	-159.59
7.955	1.796	137.637	0	-155.91
8.092	1.796	137.637	0	-154.77
8.230	1.796	137.637	0	-152.16
8.368	1.796	137.637	0	-152.22
8.505	1.796	137.637	0	-149.90
8.643	1.796	137.637	0	-149.20
8.780	1.796	137.637	0	-147.53
8.918	1.796	137.637	0	-148.45
9.056	1.796	137.637	0	-145.58
9.193	1.796	137.637	0	-146.83
9.331	1.796	137.637	0	-145.28
9.469	1.796	137.637	0	-146.40
9.606	1.796	137.637	0	-144.91
9.744	1.796	137.637	0	-145.07
9.882	1.796	137.637	0	-144.95
10.019	1.796	137.637	0	-144.50
10.157	1.796	137.637	0	-144.28
10.294	1.796	137.637	0	-142.36
10.432	1.796	137.637	0	-144.07
10.570	1.796	137.637	0	-142.02
10.707	1.796	137.637	0	-144.18
10.845	1.796	137.637	0	-141.98

Continued on next page

Table S6: – continued from previous page

$n_{1,c}$ /mmol	$n_{2,c}$ /mmol	$n_{1,s}$ /μmol	$n_{2,s}$ /μmol	Q /mJ
10.983	1.796	137.637	0	-144.13
11.120	1.796	137.637	0	-141.45
11.258	1.796	137.637	0	-143.73
11.396	1.796	137.637	0	-142.15
11.533	1.796	137.637	0	-143.66
11.671	1.796	137.637	0	-142.52
11.808	1.796	137.637	0	-143.55
11.946	1.796	137.637	0	-142.95
12.084	1.796	137.637	0	-143.46
12.221	1.796	137.637	0	-143.55
12.359	1.796	137.637	0	-143.05
12.497	1.796	137.637	0	-143.45
12.634	1.796	137.637	0	-142.82
12.772	1.796	137.637	0	-144.31
12.910	1.796	137.637	0	-142.47
13.047	1.796	137.637	0	-144.70
13.185	1.796	137.637	0	-142.44
13.322	1.796	137.637	0	-144.98
13.460	1.796	137.637	0	-142.90
13.598	1.796	137.637	0	-143.95
13.735	1.796	137.637	0	-143.11
13.873	1.796	137.637	0	-144.59
14.011	1.796	137.637	0	-142.62
14.148	1.796	137.637	0	-144.38

Continued on next page

Table S6: – continued from previous page

$n_{1,c}$ /mmol	$n_{2,c}$ /mmol	$n_{1,s}$ /μmol	$n_{2,s}$ /μmol	Q /mJ
14.286	1.796	137.637	0	-142.75
14.424	1.796	137.637	0	-143.93
14.561	1.796	137.637	0	-142.60
14.699	1.796	137.637	0	-143.35
14.836	1.796	137.637	0	-144.18
14.974	1.796	137.637	0	-142.37
15.112	1.796	137.637	0	-143.85
15.249	1.796	137.637	0	-142.69
15.387	1.796	137.637	0	-144.60
15.525	1.796	137.637	0	-142.35
15.662	1.796	137.637	0	-145.15
15.800	1.796	137.637	0	-142.51
15.938	1.796	137.637	0	-145.36
16.075	1.796	137.637	0	-142.48
16.213	1.796	137.637	0	-144.09
16.350	1.796	137.637	0	-142.75
10.587	1.878	137.630	6.556	-12.60
10.725	1.885	137.630	6.556	-12.07
10.862	1.891	137.630	6.556	-11.92
11.000	1.898	137.630	6.556	-11.84
11.138	1.904	137.630	6.556	-11.82
11.275	1.911	137.630	6.556	-11.70
11.413	1.917	137.630	6.556	-11.89
11.550	1.924	137.630	6.556	-11.74

Continued on next page

Table S6: – continued from previous page

$n_{1,c}$ /mmol	$n_{2,c}$ /mmol	$n_{1,s}$ /μmol	$n_{2,s}$ /μmol	Q /mJ
11.688	1.931	137.630	6.556	-11.63
11.826	1.937	137.630	6.556	-11.59
11.963	1.944	137.630	6.556	-11.57
12.101	1.950	137.630	6.556	-11.45

Table S7: Dissolution calorimetry data for system (1) H_2O + [P_{4444}][Bz] (2) at 298.15 K. n_i is the mole quantity of i in the cell (subscript c) or in the cartridge (subscript d for dispenser) and Q_{sol} is the integrated heat effect of the dissolution (taking into account heat effect of the reference cartridge).

$n_{1,c}$ /mmol	$n_{2,c}$ /mmol	$n_{1,d}$ /μmol	$n_{2,d}$ /μmol	Q_{sol} /mJ
919.293	0	0	16.452	-820.25
919.293	0.016	0	32.983	-1860.19
919.190	0	0	13.009	-863.15
919.190	0.013	0	33.745	-1786.28

Partial Radial Distribution Functions from EPSR model of [P_{4444}][Ph−tet]/H_2O neutron scattering data.

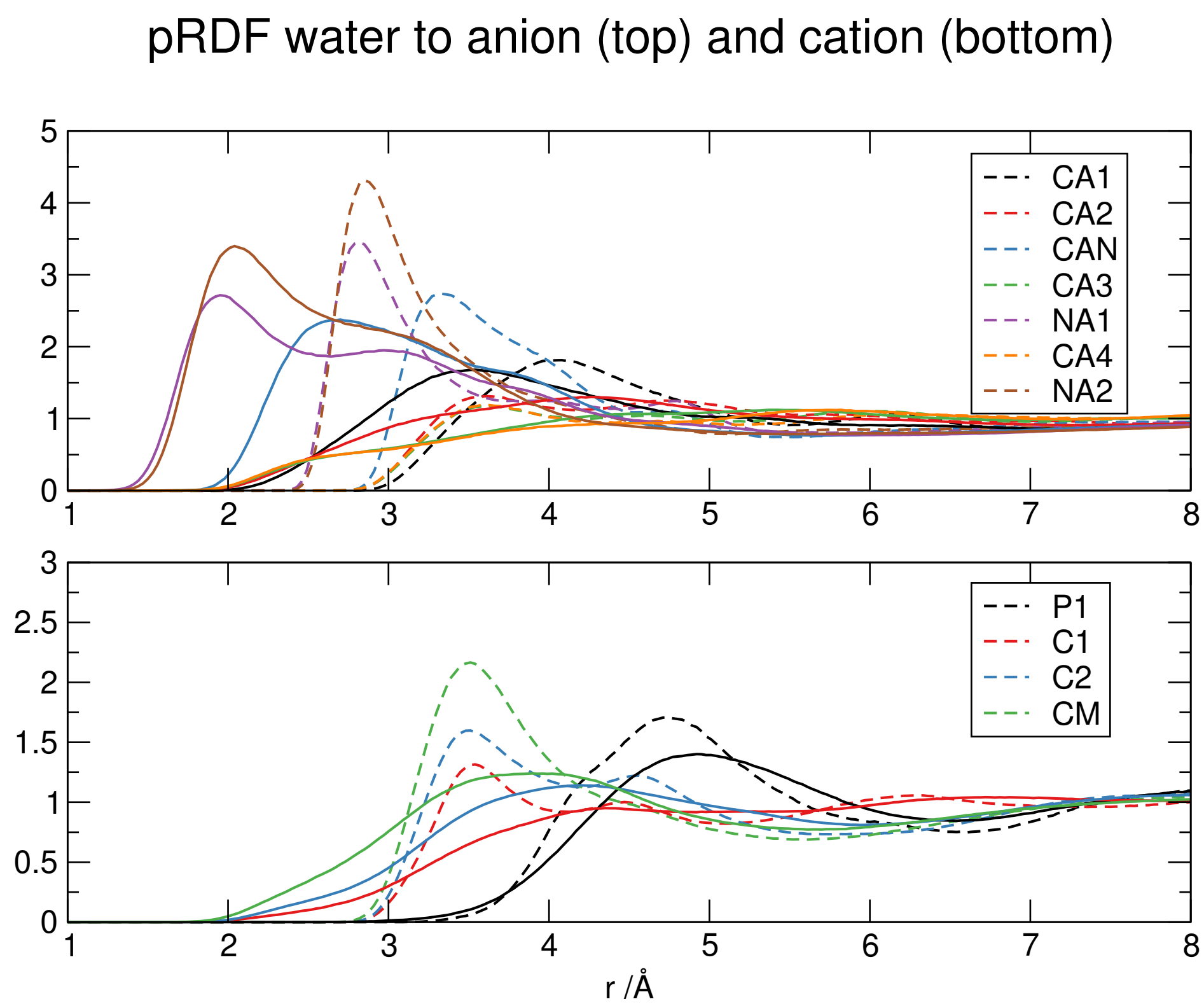

Figure S5: Site-site partial radial distribution functions between water (HW, solid lines and OW, dashed lines) and [Ph−tet]$^-$ anions (*top*) and [P_{4444}]$^+$ cations (*bottom*) showing the distinct association of water molecules through HW hydrogen-bond donation to the tetrazolate NA1/NA2 sites and significantly weaker and more diffuse contact between water OW and the phenyl ring sites (solid lines). Water association with the [P_{4444}]$^+$ is only evident through broad, non-directional contacts at the limit of VdW association (*ca*. 3.5 Å between OW and C1/C2 sites).

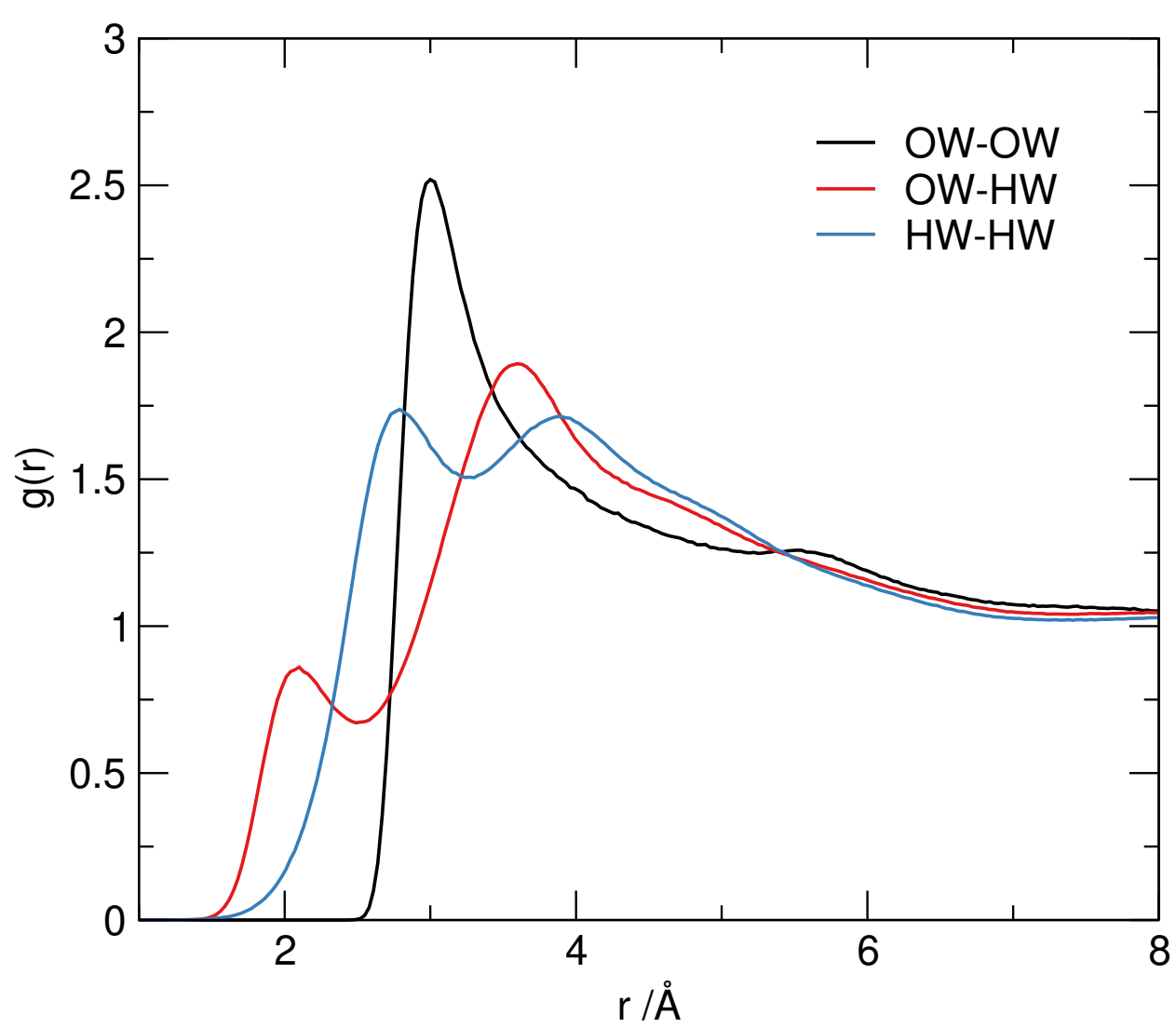

Figure S6: Water-water site-site partial radial distribution functions from the EPSR models of [P_{4444}][Ph−tet]$\cdot$5.6H_2O at 25 °C.

References

(1) Zhao, H. The synthesis and structures of deuterium-labeled 5-substituted 1H-tetrazoles. *J. Labelled Compd. Radiopharm.* **2008**, *51*, 293–296.

(2) Moura, L.; Brown, L. C.; Blesic, M.; Holbrey, J. D. LCST Phase Behavior and Complexation with Water of an Ionic Liquid Incorporating the 5-Phenyltetrazolate Anion. *ChemPhysChem* **2017**, *18*, 3384–3389.

(3) Ando, T.; Kohno, Y.; Nakamura, N.; Ohno, H. Introduction of hydrophilic groups onto the ortho-position of benzoate anions induced phase separation of the corresponding ionic liquids with water. *Chem. Commun.* **2013**, *49*, 10248–10250.

(4) Gulotty, E. M.; Sanadhya, S.; Tucker, Z. D.; Moghaddam, S. S.; Ashfeld, B. L. Controlling phase separation behavior of thermo-responsive ionic liquids through the directed distribution of anionic charge. *J. Mol. Liq.* **2022**, *360*, 119401.

(5) Yamada, T.; Park, K.; Yasukawa, N.; Morita, K.; Monguchi, Y.; Sawama, Y.; Sajiki, H. Mild and Direct Multiple Deuterium-Labeling of Saturated Fatty Acids. *Adv. Synth. Catal.* **2016**, *358*, 3277–3282.

(6) Sawama, Y.; Yamada, T.; Yabe, Y.; Morita, K.; Shibata, K.; Shigetsura, M.; Monguchi, Y.; Sajiki, H. Platinum on Carbon-Catalyzed H–D Exchange Reaction of Aromatic Nuclei due to Isopropyl Alcohol-Mediated Self- Activation of Platinum Metal in Deuterium Oxide. *Adv. Synth. Catal.* **2013**, *355*, 1529–1534.

(7) Sawama, Y.; Matsuda, T.; Moriyama, S.; Ban, K.; Fujioka, H.; Kamiya, M.; Shou, J.; Ozeki, Y.; Akai, S.; Sajiki, H. Unprecedented Regioselective Deuterium-Incorporation of Alkyltrimethylammonium Chlorides and Raman Analysis. *Asian J. Org. Chem.* **2023**, *12*, e202200710.

(8) Akutsu-Suyama, K.; Ueda, M.; Shibayama, M.; Ishii, K.; Nishi, N. Effective Synthesis of Deuterated n-Octylamine and Its Analogues. *EPJ Web of Conferences* **2023**, *286*, 01004.

(9) Tatoueix, K.; Lepron, M.; Barboux, C.; Scherrmann, M.-C.; Pieters, G.; Feuillastre, S. Unlocking the potential of hydrogen deuterium exchange via an iterative continuous-flow deuteration process. *Nat. Commun.* **2025**, *16*.

(10) Park, K.; Ito, N.; Yamada, T.; Sajiki, H. Efficient Continuous-Flow H–D Exchange Reaction of Aromatic Nuclei in D_2O/2-PrOH Mixed Solvent in a Catalyst Cartridge Packed with Platinum on Carbon Beads. *Bull. Chem. Soc. Jpn.* **2020**, *94*, 600–605.

(11) Levernier, E.; Tatoueix, K.; Garcia-Argote, S.; Pfeifer, V.; Kiesling, R.; Gravel, E.; Feuillastre, S.; Pieters, G. Easy-to-Implement Hydrogen Isotope Exchange for the Labeling of N-Heterocycles, Alkylkamines, Benzylic Scaffolds, and Pharmaceuticals. *JACS Au* **2022**, *2*, 801–808.

(12) McGrogan, A.; Lafferty, J.; O'Neill, L.; Brown, L.; Young, J. M.; Goodrich, P.; Muldoon, M. J.; Moura, L.; Youngs, S.; Hughes, T.-L. et al. Liquid Structure of Ionic Liquids with $[NTf_2]^-$ Anions, Derived from Neutron Scattering. *J. Phys. Chem. B* **2024**, *128*, 3220–3235.

(13) (a) Westheimer, F. H. The Magnitude of the Primary Kinetic Isotope Effect for Compounds of Hydrogen and Deuterium. *Chem. Rev.* **1961**, *61*, 265–273; (b) Sharp, T. E.; Johnston, H. S. Hydrogen-Deuterium Kinetic Isotope Effect, an Experimental and Theoretical Study over a Wide Range of Temperature. *J. Chem. Phys.* **1962**, *37*, 1541–1553; (c) Simmons, E. M.; Hartwig, J. F. On the Interpretation of Deuterium Kinetic Isotope Effects in C-H Bond Functionalizations by Transition-Metal Complexes. *Angew. Chem. - Int. Ed.* **2012**, *51*, 3066–3072.

(14) Yamada, T.; Sawama, Y.; Shibata, K.; Morita, K.; Monguchi, Y.; Sajiki, H. Multiple

deuteration of alkanes synergistically-catalyzed by platinum and rhodium on carbon as a mixed catalytic system. *RSC Advances* **2015**, *5*, 13727–13732.
